# Inferring Invasion History of Red Swamp Crayfish (*Procambarus clarkii*) in China from Mitochondrial Control Region and Nuclear Intron Sequences

**DOI:** 10.3390/ijms160714623

**Published:** 2015-06-29

**Authors:** Yanhe Li, Xianwu Guo, Liping Chen, Xiaohui Bai, Xinlan Wei, Xiaoyun Zhou, Songqian Huang, Weimin Wang

**Affiliations:** 1 Key Laboratory of Agricultural Animal Genetics, Breeding and Reproduction of Ministry of Education, College of Fisheries, Huazhong Agricultural University, Wuhan 430070, China; E-Mails: yanhel6@126.com (Y.L.); chenliping@webmail.hzau.edu.cn (L.C.); bxh123bxh@163.com (X.B.); weixinlan813@163.com (X.W.); zhouxy@mail.hzau.edu.cn (X.Z.); huangsongqian@163.com (S.H.); 2 Institute of Fisheries, Anhui Academy of Agricultural Sciences, Hefei 230031, China; 3 Laboratorio de Biotecnología Genómica, Centro de Biotecnología Genómica, Instituto Politécnico Nacional, Boulevard del Maestro esquina Elías Piña, Colonia Narciso Mendoza, Ciudad Reynosa 88710, Tamaulipas, Mexico; E-Mail: gxianwu@yahoo.com; 4 Tianjin Fisheries Research Institute, Tianjin 300221, China

**Keywords:** *Procambarus clarkii*, mitochondrial control region, intron, Approximate Bayesian Computation, invasion

## Abstract

Identifying the dispersal pathways of an invasive species is useful for adopting the appropriate strategies to prevent and control its spread. However, these processes are exceedingly complex. So, it is necessary to apply new technology and collect representative samples for analysis. This study used Approximate Bayesian Computation (ABC) in combination with traditional genetic tools to examine extensive sample data and historical records to infer the invasion history of the red swamp crayfish, *Procambarus clarkii*, in China. The sequences of the mitochondrial control region and the *proPOx* intron in the nuclear genome of samples from 37 sites (35 in China and one each in Japan and the USA) were analyzed. The results of combined scenarios testing and historical records revealed a much more complex invasion history in China than previously believed. *P. clarkii* was most likely originally introduced into China from Japan from an unsampled source, and the species then expanded its range primarily into the middle and lower reaches and, to a lesser extent, into the upper reaches of the Changjiang River in China. No transfer was observed from the upper reaches to the middle and lower reaches of the Changjiang River. Human-mediated jump dispersal was an important dispersal pathway for *P. clarkii*. The results provide a better understanding of the evolutionary scenarios involved in the rapid invasion of *P. clarkii* in China*.*

## 1. Introduction

Introduced species exist in almost every ecosystem in the world, and the proportions of introduced species in biomes, ecosystems, and habitats are increasing [1]. Invasive species are of concern because they represent a major threat to the biodiversity of their new habitats [2] and could have serious implications for human well-being, and a strong influence on local economies [3,4]. As awareness of the complexity of the problems of invasive species has gradually increased, invasion ecology, including invasive species population structure, genetic diversity and evolutionary histories, has evolved in response to challenges in biodiversity conservation and invasion control [1]. Thus, information regarding changes in the population structure, genetic diversity and evolutionary histories of invasive species, such as retracing the dispersal routes and identification of source populations, would be useful to establish possible methodologies for the prevention and control of their invasions [5].

Source populations and pathways of invasion can be identified by either direct or indirect methods [6,7,8]. Indirect methods are primarily based on molecular and statistical analyses of invasive species populations collected from both source and invaded areas, whereas direct methods are almost always based on historical data [6,8]. However, sometimes the historical data are generally so ambiguous or incomplete that it is difficult to identify the routes of invasion for an invasive species. A successful invasion may not even occur despite a historical record of an introduction [8]. Indirect molecular methods have been commonly used to investigate the origin and dispersal of an invasive species by investigating its population structure and comparing the genetic diversity and relatedness among populations [7,9]. In general, the molecular markers used for population genetics and phylogeography research, such as nuclear introns and the mitochondrial control region, are also appropriate for investigating the invasion history and pathways of a species.

Approximate Bayesian Computation (ABC) testing, a recently developed method [10,11], has provided a method to determine the invasion routes of introduced species using microsatellite data [8,12] and/or DNA sequences. Five categories of loci (either microsatellites or DNA sequences) can now be analyzed together or separately for species invasion research using the DIYABC v1.0.4.46 beta which is a user-friendly approach to ABC for inference on population history using molecular markers [11]. The new developments of the integrated DIYABC software are particularly useful in understanding complex evolutionary scenarios involving both recent and ancient historical events [11].

*Procambarus clarkii* (Girard, 1852), the red swamp crayfish, is one of the most notorious invasive species in the world [13,14]. It invaded China in the early 20th century and is now found in almost all forms of freshwater bodies, including lakes, rivers and even paddy fields, in most Chinese provinces [15,16]. The crayfish threaten local biodiversity in freshwater ecosystems, and their burrows result in dam damages and a huge loss of irrigation water, which causes significant economic loss [17,18]. A high level of genetic diversity was detected in Chinese populations of *P. clarkii* [9,14,15], and some individuals from sampling sites in China tended to be more admixed, indicating that multiple invasions may have occurred [9]. Generally, factors such as genetic variations of multiple introductions, hybridization and adaptation, which occur after successfully invading a new environment, might cause the high genetic diversity of an invasive species [19]. The successful invasion of *P. clarkii* in China could be the consequence of human-mediated dispersal [9,20] and adaptive variation [19] in addition to multiple introductions and/or other unintentional introductions [9].

As we know, the mitochondrial control region is the most variable part of the mitochondrial genome and evolves three to five times more rapidly than does the rest of the mitochondrial genome [21]. It is generally suitable for analyzing evolution, population genetic diversity and phylogenetic relationships among intraspecific populations. Furthermore, unlike exons, the intron sequences appear to be subject to little functional constraint and accumulates mutations rapidly, displaying high genetic variation [22,23]. Powerful analyses of population structure and related studies require information from multiple genetic loci [24]. In a previous study, we investigated the population genetic structure and post-establishment dispersal patterns of *P. clarkii* using mitochondrial cytochrome c oxidase I (*COI*), 16S ribosomal RNA (*rRNA*) and nuclear microsatellites data sets [9], and found that the crayfish founder population in China might have been derived from Japan. The *P. clarkii* populations in China have a relatively high genetic diversity. The aim of the present study was to investigate the routes of invasion of *P. clarkii* in China. The sequences of the mitochondrial control region and the *proPOx* intron were used to assess the genetic diversity and variation of the population and to analyze networks of their haplotypes. In addition, the mixed mitochondrial control region and *proPOx* intron sequence data were analyzed using ABC to test statistically distinct scenarios based on the clusters of *P. clarkii* population. The expected results were (i) detection of variations in the control region and the *proPOx* intron; (ii) generation of networks of *P. clarkii* haplotypes and (iii) inference of scenarios and routes of invasion.

## 2. Results

### 2.1. Genetic Diversity and Haplotypes

**Figure 1:**
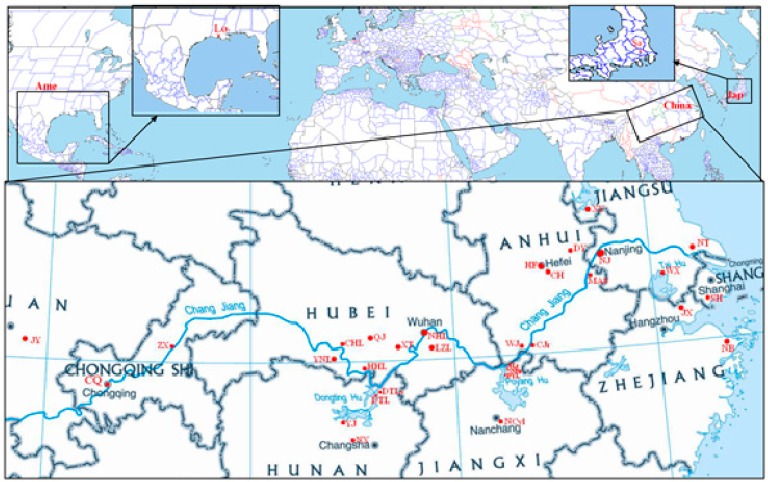
Sampling locations of *P. clarkii*. The lower panel shows an enlarged view of the sampling locations in China. Red dots indicate the sampling locations. The blue line on the map of China denotes the Changjiang River. XY includes the XYw and XYc population. NJ includes the XG, BGt and XBv populations. WX includes the WX and WXb populations. For the sampling location codes, see Table 1.

Based on the data from the mitochondrial control region sequences (291 individuals from 37 sites as shown in Figure 1 and Table 1), the nucleotide diversity was 0.01031. Forty-six haplotypes (GenBank accession numbers: KC556828–KC556873) were found, and their distributions are presented in Figure S1. The haplotype diversity was 0.498, and the variance and standard deviation of haplotype diversity were 0.00100 and 0.032, respectively. Under the Maximum Composite Likelihood model, the overall mean pairwise genetic distance of the 46 haplotypes was 0.016. From the analysis of the 196 *proPOx* intron sequences, the nucleotide diversity was 0.00640. Nine haplotypes (GenBank accession numbers: KC556874–KC556882) were found, and their distributions are presented in Figure S1. The haplotype diversity, the variance of haplotype diversity and their standard deviation were 0.664, 0.00032 and 0.018, respectively. Using the Maximum Composite Likelihood model, the overall mean pairwise genetic distance of the nine haplotypes was 0.019.

**Figure 2:**
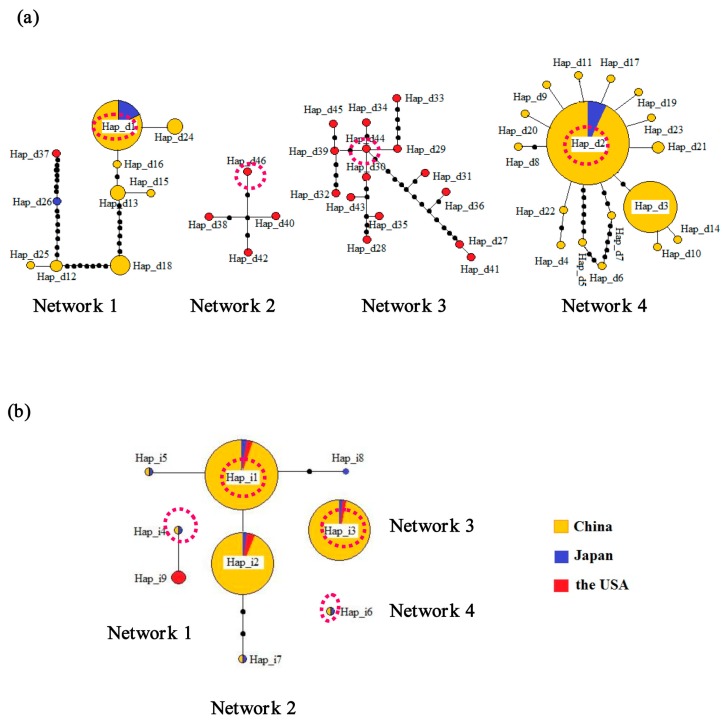
Statistical parsimony networks of the control regions (**a**) and *proPOx* intron sequences (**b**) for the *P. clarkii* samples. The small black dots represent the missing haplotypes. The ancestral haplotypes for each network lie within the broken red line. The haplotypes are shown in different colors according to the geographical regions from which the samples were collected. The circle size is proportional to the observed haplotype frequency.

As regards the mitochondrial control region, the 291 individual samples comprised 46 unique haplotypes, and the statistical parsimony analysis in TCS (which is a software to estimate phylogenetic network using statistical parsimony) revealed four distinct networks (Figure 2a). Four haplotypes (Hap_d1, Hap_d2, Hap_d44, and Hap_d46) were inferred to be ancestral because they yielded the highest outgroup weights in each of the networks (0.24, 0.40, 0.20 and 0.25, respectively) [25]. Network 1 contained 10 haplotypes found in the samples collected from the USA, Japan and China. Network 2 included four haplotypes and Network 3 included 15 haplotypes, all of which only occurred in the samples collected in the USA. Network 4 contained 17 haplotypes found in the individuals from Japan and China (Figure 2a and Figure S1). In addition, Network 4 was characterized by a closed loop instead of a linear relationship connecting haplotypes, suggesting the presence of homoplasy [26]. The 196 specimens examined using the *proPOx* intron sequences comprised nine unique haplotypes, and the statistical parsimony analysis revealed four distinct networks and two haplotypes (Networks 3 and 4) that could not be connected with a confidence limit of 95% [26] (Figure 2b). Four haplotypes (Hap_i1, Hap_i3, Hap_i4, and Hap_i6) were inferred to be ancestral because they yielded the highest outgroup weights in each of the networks (0.46, 1.00, 0.50 and 1.00, respectively) [25]. Network 1 included two haplotypes that were found in samples from the USA, Japan and China (Figure 2b and Figure S1). Network 2 contained five haplotypes that were found in the populations collected from the USA, Japan and China. Network 3 also contained only haplotype Hap_i3, found in the American, Japanese and Chinese populations. Network 4 only contained the haplotype Hap_i6, found in one Chinese population (population QJ) and the Japanese population.

**Table 1:** List of the populations of *P. clarkii* studied, indicating their location and country of origin, geographical position of sampling site, and haplotype diversity of the mitochondrial control region and *proPOx* intron sequences.

Code	Location (Country)	Longitude	Latitude	Control Region Sequencing			*ProPOx* Intron Sequencing		
				Control Region (*N*/*H*)	*H*_d_	*P*_i_	*ProPOx* Intron (*N*/*H*)	*H*_d_	*P*_i_
SH	Shanghai (China)	121.23 °E	31.03 °N	(7/2)	0.476	0.0121	(3/3)	1.000	0.0108
NB	Ningbo (China)	121.55 °E	29.88 °N	(8/1)	0.000	0.0000	(4/1)	0.000	0.0000
JX	Jiaxing (China)	120.77 °E	30.75 °N	(7/2)	0.286	0.0072	(2/1)	0.000	0.0000
XYc	Xuyi-culture (China)	118.50 °E	33.00 °N	(7/4)	0.714	0.0055	(6/2)	0.600	0.0097
XYw	Xuyi-wild (China)	118.42 °E	33.03 °N	(7/2)	0.286	0.0003	(4/2)	0.500	0.0020
WXb	Binhu, Wuxi (China)	120.28 °E	31.52 °N	(9/2)	0.500	0.0013	(6/3)	0.733	0.0062
NT	Nantong (China)	120.87 °E	32.02 °N	(7/2)	0.286	0.0073	(6/2)	0.533	0.0065
XG	Xiaguan district (Nanjing, China)	118.75 °E	32.08 °N	(8/1)	0.000	0.0000	(7/2)	0.476	0.0019
XBv	Xiaba village (Nanjing, China)	118.87 °E	32.20 °N	(7/1)	0.000	0.0000	(6/2)	0.333	0.0014
BGt	Baguazhou township (Nanjing, China)	118.82 °E	32.17 °N	(7/2)	0.286	0.0003	(7/2)	0.476	0.0019
WX	Wuxi (China)	120.30 °E	31.57 °N	(6/2)	0.533	0.0135	(7/3)	0.714	0.0081
WJ	Wangjiang (China)	116.70 °E	30.12 °N	(7/1)	0.000	0.0000	(8/2)	0.536	0.0022
MAS	Maanshan (China)	118.50 °E	31.55 °N	(8/4)	0.643	0.0114	(6/2)	0.333	0.0041
CJr	Guangfengwei section of Changjiang river (China)	116.87 °E	30.12 °N	(7/2)	0.286	0.0003	(7/2)	0.476	0.0019
CH	Chaohu (China)	117.87 °E	31.62 °N	(7/2)	0.476	0.0121	(7/2)	0.476	0.0019
HF	Hefei (China)	117.23 °E	31.82 °N	(8/2)	0.250	0.0063	(6/2)	0.333	0.0014
DY	Dingyuan (China)	117.83 °E	32.28 °N	(6/2)	0.600	0.0152	(3/1)	0.000	0.0000
SLt	Sanli township (China)	116.22 °E	29.75 °N	(8/3)	0.607	0.0111	(5/1)	0.000	0.0000
NBp	Nanbei Port (China)	116.17 °E	29.72 °N	(8/1)	0.000	0.0000	(7/3)	0.524	0.0023
PYL	Poyang lake (China)	116.43 °E	28.87 °N	(7/1)	0.000	0.0000	(2/2)	1.000	0.0041
NCyl	Youlan, Nanchang (China)	116.12 °E	28.52 °N	(8/1)	0.000	0.0000	(3/1)	0.000	0.0000
NHL	Nanhu lake (China)	114.03 °E	30.02 °N	(7/1)	0.000	0.0000	(4/1)	0.000	0.0000
YNL	Yuni lake (China)	112.20 °E	30.00 °N	(8/1)	0.000	0.0000	(6/2)	0.600	0.0024
XT	Xiantao (China)	113.40 °E	30.30 °N	(8/1)	0.000	0.0000	(6/2)	0.600	0.0024
QJ	Qianjiang (China)	112.60 °E	30.40 °N	(7/1)	0.000	0.0000	(4/2)	0.500	0.0182
LZL	Liangzi lake (China)	114.00 °E	30.00 °N	(6/1)	0.000	0.0000	(2/2)	1.000	0.0041
HHL	Honghu lake (China)	113.40 °E	29.70 °N	(8/2)	0.250	0.0003	(2/2)	1.000	0.0122
CHL	Changhu lake (China)	112.10 °E	30.30 °N	(7/1)	0.000	0.0000	(6/3)	0.733	0.0062
YJ	Yuanjiang (China)	112.37 °E	28.85 °N	(7/1)	0.000	0.0000	(5/3)	0.700	0.0089
NX	Ningxiang (China)	112.55 °E	28.28 °N	(8/1)	0.000	0.0000	(4/3)	0.833	0.0081
DTL	Dongting lake (China)	113.02 °E	29.30 °N	(6/1)	0.000	0.0000	(2/2)	1.000	0.0122
DTLs	Dongting lakeside (China)	113.13 °E	29.35 °N	(8/2)	0.250	0.0003	(4/2)	0.500	0.0061
CQs	Chongqing suburb (China)	106.53 °E	29.55 °N	(7/1)	0.000	0.0000	(8/2)	0.429	0.0017
ZX	Zhongxian (China)	108.03 °E	30.28 °N	(6/2)	0.533	0.0141	(7/3)	0.667	0.0058
JY	Jianyang (China)	104.55 °E	30.38 °N	(6/2)	0.333	0.0088	(6/2)	0.533	0.0022
Sa	Saitama (Japan)	139.65 °E	35.85 °N	(18/3)	0.569	0.0132	(9/7)	0.917	0.0180
LA	Louisiana (USA)	93.26 °W	29.87 °N	(20/16)	0.963	0.0086	(9/4)	0.778	0.0065

*N* = sample size; *H* = number of haplotypes; *H*_d_ = haplotype diversity; and *P*_i_ = nucleotide diversity.

### 2.2. Variation and Genetic Structure

For the mitochondrial control region, a total of 65 variable (polymorphic) sites and 43 sites with alignment gaps were observed. Of the polymorphisms, 14 were singleton variable sites of two variants, 49 were parsimony informative sites of two variants, and two were parsimony informative sites of three variants (Figure S2). For the *proPOx* intron, we observed 16 variable (polymorphic) sites and 21 sites with alignment gaps. Of the polymorphisms, two were singleton variable sites of two variants, ten were parsimony informative sites of two variants, and four were parsimony informative sites of three variants (Figure S2).

Neither Tajima’s *D* nor *Fu* and *Li*’s D* rejected the null hypothesis of neutral evolution (mitochondrial control region, *D* = −0.26683, D* = −1.25495; *p* > 0.10; *proPOx* intron, *D* = −1.44130, D* = 0.72984; *p* > 0.10); therefore, variation patterns of the mitochondrial control region and the *proPOx* intron sequences may be a reliable reflection of the population history of *P. clarkii*. Moreover, the positive *Fs* values derived from the mitochondrial control region sequence and the *proPOx* intron sequence analyses were 0.773 (*p* = 0.393 > 0.05) and 0.365 (*p* = 0.572 > 0.05), respectively, indicating that *P. clarkii* did not experience a significant population expansion.

The AMOVA revealed that high levels of variations in the mitochondrial control region and the *proPOx* intron were found both among and within populations (Table S1). The AMOVA of the mitochondrial control region sequences analysis revealed that 45.91% of the genetic variations could be explained by the variations within a population, whereas the remaining variations (54.09%) came from variations among the populations. The AMOVA of the *proPOx* intron sequences analysis revealed that the percentage of variations found within populations was 69.55%, whereas the remaining 30.45% were attributed to variations among the populations.

The Mantel test results for matrix correlation between genetic similarity and geographical distances were not significant (mitochondrial control region sequences, *r* = −0.1031, *p* = 0.1240; *proPOx* intron sequences, *r* = −0.0760, *p* = 0.2170). Thus, no pattern of IBD was shown for the crayfish in China.

### 2.3. Scenario Testing

**Figure 3:**
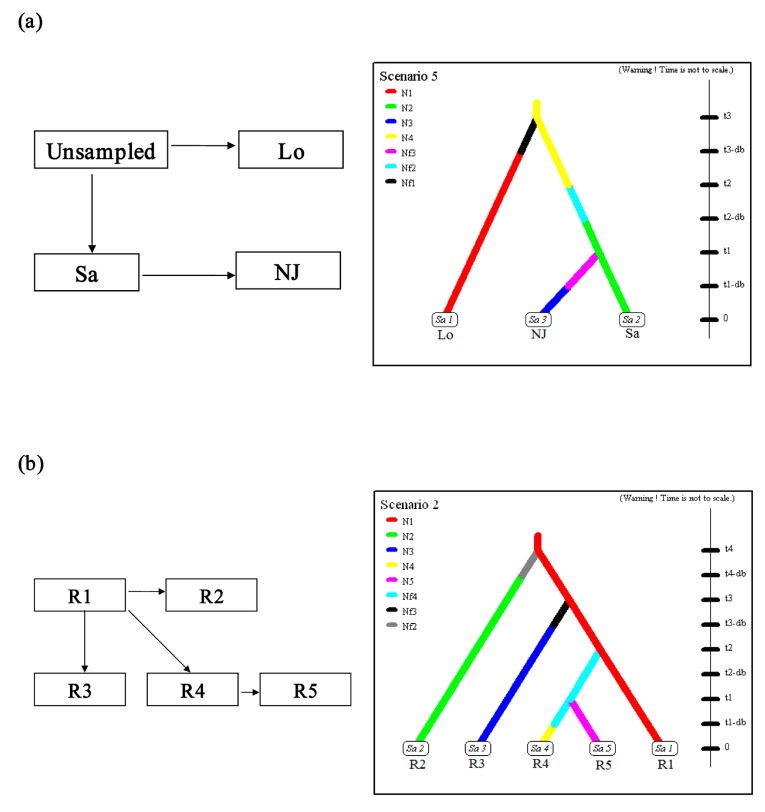
Historical scenarios supported by highest posterior probabilities in analyses using Approximate Bayesian Computation and schematic representation of the competing scenarios considered for the inference of the invasion routes. (**a**) Historical scenario supported by highest posterior probabilities and its schematic representation for cluster 1; (**b**) Historical scenario supported by highest posterior probabilities and its schematic representation for cluster 2. The values of the sample sizes that were measured in number of individuals were *N* and *Nf*. The values of times that were measured in number of generations were *db* and *t*. The populations (Lo, Sa and NJ) with effective populations sizes *N1*, *N2* and *N3*, at time/generations *t3*-*db*, *t2*-*db* and *t1*-*db*, diverged from ancestral populations with effective populations sizes *Nf1*, *Nf2* and *Nf3*, which were from an population with effective population size of *N4* at time/generations *t3*, *t2* and *t1* respectively. R1 contained the sampling populations XYw, XYc, XG, BGt, XBv, DY, MAS, HF and CH; R2 included JX, ZX and CQ; R3 included YNL, CHL, QJ, NHL, LZL, XT, HHL, DTLs, DTL, YJ and NX; R4 contained WJ, CJr, SLt, NBp, PYL and NCyL; R5 included WX, WXb, NT, JX, SH and NB. The populations (R2, R3, R4 and R5) with effective populations sizes *N2*, *N3*, *N4*, and *N5*, at time/generations *t4*-*db*, *t3*-*db*, *t1* and *t1*-*db*, diverged from ancestral populations with effective populations sizes *Nf2*, *Nf3* and *Nf4* which were from an population with effective population size of *N1* at time/generations *t4*, *t3* and *t2* respectively.

In the scenario testing analyses, different competing invasion scenarios were compared, and the parameters for the most probable scenarios were estimated using ABC. The different scenarios considered are represented in Figure S3. The best supported scenarios are shown in Figure 3, and the prior distributions are represented in Table S2.

Nanjing, Jiangsu province is the presumed initial entry point in China [9,16]; thus, the populations La (from Louisiana, USA), Sa (from Saitama, Japan) and NJ (three populations of Nanjing in China combined) were designated as cluster 1. Based on the historical data indicating that the south-central USA was the origin of *P. clarkii* [13] and that the crayfish in Japan were introduced from the USA as feed [27], six scenarios (scenarios 1–6) were considered (Figure S3). Scenario 5, which assumes that the *P. clarkii* in Japan originated via introductions from an unsampled source followed by serial introductions from Japan into NJ, was the most strongly supported scenario (PP: 0.69; 95% confidence interval CI: 0.28–1.00; Table S2, Figure 3a and Figure S3). For these scenarios, the type I error rate was substantial (0.712), but the type II error rate was relatively small (0.172).

**Figure 4:**
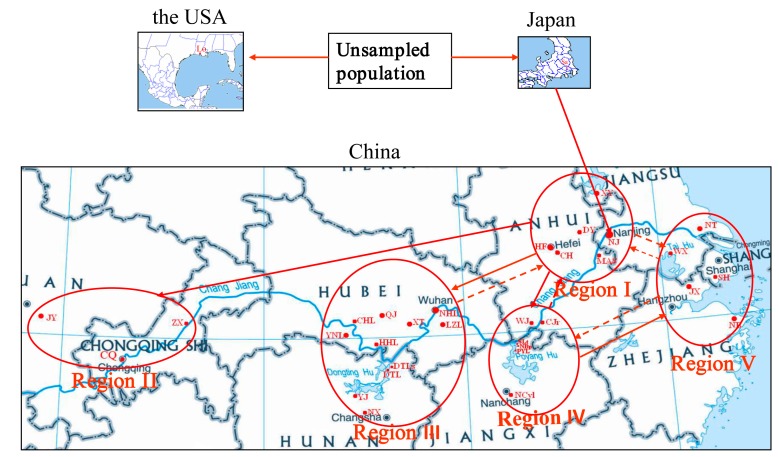
Summary of the Bayesian clustering analyses per region and schematization of the dispersal of *P. clarkii* inferred from this study. The red circles represent the populations/regions for cluster 2 tested using DIYABC. Region I included the sampling sites/populations XY (XYw and XYc), NJ (XG, BGt and XBv), DY, MAS, HF and CH; Region II included JX, ZX and CQ; Region III contained YNL, CHL, QJ, NHL, LZL, XT, HHL, DTLs, DTL, YJ and NX; Region IV included WJ, CJr, SLt, NBp, PYL and NCyL; Region V contained WX, WXb, NT, JX, SH and NB. The red solid arrow indicated that the transfer was highly supported based on the molecular data in this study. The red dotted arrows indicated that maybe there were reverse/mutual transfers between regions while the dotted arrows of opposite direction between Region I and V showed that multiple mutual transfers maybe existed between them. The blue line on the maps of China denotes the Changjiang River.

The samples collected in China (excluding the more admixture populations) were classified into five populations (regions) based on the distribution of the Chinese populations (Figure 1) and the data analyses in our previous study [9] and included in cluster 2 (Figure 4). Five different scenarios (scenario 7–11) were eventually considered based on the known historical dates of the first record of *P. clarkii* in China (Figure S3) and the STRUCTURE algorithm results and hypotheses presented in our previous study [9]. Scenario 8, which assumes that the most strongly supported scenario involved Region I as the origin of the populations of Regions II–IV with subsequent serial introduction from Region IV into V (PP: 0.47; 95% CI: 0.03–0.91; Table S2; Figure S3 and Figure 3b). For these scenarios, the type I error rate (0.178) and the type II error (0.158) were both low.

Finally, a total of 19 scenarios (#12–30) were included in cluster 3 (Figure S3). However, no scenario had a higher posterior probability; thus, power analyses were not performed for these scenarios.

Taken together, the inferred routes of invasion resulting from scenario testing are summarized in Figure 4. The crayfish invaded China from Japan and then mainly expanded and transferred into the middle and lower reaches of the Changjiang River in China. Additionally, we investigated whether *P. clarkii* was introduced from the middle and lower reaches of the Changjiang River into the river’s middle and upper reaches, but we did not find evidence for such a transfer.

## 3. Discussion

With the recent developments in population genetic approaches, researchers can infer the origin, pathways of introduction, mode of establishment and demographic changes associated with the dispersal of invasive species in invaded areas based on information about the population structure and the past demography of populations; such approaches can be combined with traditional genetic tools and historical records [8]. Our study has taken advantage of these integrated methods to infer the invasion history of *P. clarkii* in China.

The six ancestral haplotypes (Hap_d1, Hap_d2, Hap_i1, Hap_i3, Hap_i4, and Hap_i6) detected in various populations in China were shared by the populations in Japan and China. Only the Hap_i1 and Hap_i3 were shared by the populations from the USA, Japan and China. Therefore, Japan rather than the USA was the probable origin of Chinese crayfish populations. The scenario testing for cluster 1 revealed that an unsampled source population was most likely involved in the overall genetic make-up, which meant that some genetic resource of *P. clarkii* populations in China might come from unsampled place(s) completely different from the foreign populations collected in this study. It indicated that (i) insufficient source populations were sampled or/and that (ii) Japan introduced *P. clarkii* multiple times or from multiple places including unexpected places. Besides, of course, genetic variations may have occurred in Chinese populations after invasion, which maybe to some extent contributed to the unsampled source. The unsampled population may to some extent contribute to the ancestral haplotypes detected by the network analyses. Undoubtedly, further sampling in native areas would help to clarify the source area of the genetic clusters. Regardless, the scenario testing for cluster 1 also indicated that the most strongly supported scenario involved introduction routes for the crayfish invading China from Japan. The results were consistent with those obtained from microsatellites, mitochondrial *COI* and *16S* rRNA sequences in our previous study, which suggested that the *P. clarkii* populations in China might be derived from Japan rather than the USA [9]. More samples from possible original regions including USA and Japan, and more compelling evidence with further data analyses would be needed to elucidate actual sources and further details of invasion routes.

The most likely scenario regarding the invasion of cluster 1 and the most strongly supported scenario for the invasion of cluster 2 revealed an unsampled source followed by a serial introduction from Japan into Nanjing, Jiangsu province, China. The original population was then mainly expanded and transferred to other areas in the Changjiang River basin in China. These results are consistent with those previously reported in the literature [9,16]. However, interestingly, we found that *P. clarkii* was introduced from the middle and lower reaches of the Changjiang River into the river’s middle and upper reaches, but no converse transfer was observed. This finding may be resulted from (i) the crayfish habit of swimming against the flow and (ii) that the number of crayfish naturally distributed in the middle and upper reaches of the Changjiang River was very small, and thus, the crayfish was unlikely to be transferred by humans due to commerce. Thus, the results also indicated that human-mediated dispersal was an important dispersal pathway for *P. clarkii* in China, and the results to some extent showed that the aim of commerce might mostly contribute to the human-mediated dispersal for the crayfish. The human-mediated dispersal might contribute to the complex of dispersal pathways and the gene flows among the populations in the middle and lower reaches of the Changjiang River.

Scenario testing for cluster 3, exclusively analyzing the admixture populations, showed that no scenario had higher posterior probability. *P. clarkii* is an economically important aquaculture species in China, and frequent aquaculture-related transfers have occurred among different locations. Human activities such as commercial shipping or unintentional carry have most likely facilitated gene flow among populations of *P. clarkii* and influenced their population genetic structure and diversity [16]. The transfers were so frequent that there were complex routes and multiple introductions rather than a certain route or a single independent introduction for the crayfish invasion among these regions. Thus, the scenario testing results of cluster 3 presented here have most likely resulted from very frequent human-mediated transfers among the regions observed. The results of this study also showed that the dispersal pathways for *P. clarkii* might be much more complex than previously assumed.

Generally, increased genetic diversity and/or multiple introductions could contribute to invasion success [28]. However, evolution and adaptation also played an important role in this invasion process [9,29]. It has been demonstrated that putatively adaptive traits have evolved in introduced populations, occasionally quite rapidly [30,31]. In our study, AMOVA showed that many genetic variations occurred within the samples of a *P. clarkii* population and between population samples, which, to some extent, might have enhanced the invasion success of *P. clarkii*. The results are similar to those obtained in a previous study in which AMOVA also showed that many genetic variations occurred among and within the *P. clarkii* populations based on *COI*, *16S* rRNA and nuclear microsatellites data sets [9]. Furthermore, different derived haplotypes were detected in the Chinese populations, possibly indicating new variations occurring among the specimens in China. However, further investigation focusing on adaptive evolution, molecular data from *P. clarkii* from the source and invasive areas, and application of additional methodologies, particularly those related to evaluating the evolutionary history of *P. clarkii* invasion, is needed.

Biological invasions could pose a threat to agriculture, natural environments, and human economic activities [32]. Presently, the pace of the invasion process has been accelerated by globalization [33], and biological invasions are a significant component of global environmental change [32,34]. Understanding geographical pathways of invasive species populations could clarify the origin and genetic structure of such populations and could help develop management strategies to quarantine, control or eradicate an invading species [32]. Thus, in this study, the results of *P. clarkii* invasion path identification could help support our understanding of why the crayfish mainly distributed in the middle and lower reaches, and further indicate that human-mediated dispersal was one of the important dispersal pathways for *P. clarkii* in China. It could also help quarantine, control or eradicate *P. clarkii* in China, and provide a reference for scientists to introduce a new species in the future.

## 4. Materials and Methods

### 4.1. Sampling

A total of 35 sites were selected in China, and one site each from the USA and Japan were selected (Figure 1 and Table 1). The 35 sites in China were focused on the areas of major cultivation and major river basins where *P. clarkii* distributed in China, and the USA and Japan sites are the alleged initial places of introduction. The Chinese sites were located in open, abandoned fields, and no specific permits were required for the described field studies. The samples from the sites in Japan and the USA were kindly collected by Dr. Jian Gao from Kagoshima University and Dr. Dan Wang from Ohio State University South Centers, respectively, with permission from the property owners. The muscle samples were dissected from the crayfish and were immediately stored in 95% ethanol. After returning to the laboratory, the samples were stored at −20 °С for later DNA extraction.

### 4.2. DNA Isolation, Amplification and Sequencing

Genomic DNA was isolated using a method adapted from Li *et al.* [35]. The primers (*CR*F: 5′-TCGCTGTAAAGTTGAAGAAGTT-3′; *CR*R: 5′-TTAATCTCTTCATATCTTTAATTAC-3′) were used to amplify ~957–983 bp of the control region in 50 µL polymerase chain reactions (PCRs). The reaction conditions were as follows: 5 cycles of denaturation at 95 °C for 30 s, annealing at 52 °C for 1 min, and elongation at 72 °C for 1 min, followed by 30 cycles of denaturation at 95 °C for 30 s, annealing at 50 °C for 1 min, and elongation at 72 °C for 1 min, and a final 10-min extension at 72 °C and cooling to 4 °C. The PCR products were sent directly to Sangon Biotech Co., Ltd. (Shanghai, China) for purification and sequencing using the forward PCR primer *CR*F and an internal forward sequencing primer *CR*F2-F (5′-TCAAGAATATGATAGTAATGTT-3′).

An intron of the gene encoding prophenoloxidase (*proPO*) of *P. clarkii*, referred to as the *proPOx* intron in this study, spans a region of approximately 240–270 bp. The primers designed to amplify the complete *proPOx* intron region [36] were as follows: *proPO*xF (5′-GAGACGTAATGAGTTCGGGCTGG-3′) and *proPOx*R (5′-GCTCAGCTGACCATCTGCCTCAA-3′). The amplification reactions were performed in a volume of 50 µL using the following cycling conditions: 94 °С for 5 min, followed by 30 cycles of 94 °С for 30 s, 58 °С for 45 s and 72 °С for 30 s, with a final extension at 72 °С for 10 min. These PCR products were also sent directly to Sangon Biotech Co., Ltd. (Shanghai, China) for purification and sequencing. Due to polymorphisms in the intron, some of the PCR products were detected as “double peaks” in certain intron fragments in the chromatogram. These “double peak” PCR products were further cloned using the pGEM^®^-T Easy Vector System (Promega Corporation, Madison, WI, USA). However, the sequences obtained from these clones were contained in those haplotype sequences of corresponding populations obtained by direct sequencing. Thus, in the haplotype analysis, we omitted the *proPOx* intron sequences that appeared as “double peaks”.

### 4.3. Data Analyses

#### 4.3.1. Alignment of the Sequences

All of the sequences (including the control region sequences of 291 individuals and *proPOx* intron sequences of 196 individuals) obtained from the 37 populations were aligned using Clustal X 2.1 [37] and MEGA 5.05 [38] with the default parameters.

#### 4.3.2. Variations and Genetic Diversity

The variable sites, parsimony informative sites, number of haplotypes, nucleotide diversity, and neutrality test (Fu’s Fs, Li’s, and Tajima’s tests) were assessed using DnaSP 5.10 software [39]. Analysis of molecular variance (AMOVA) was conducted using ARLEQUIN v3.5.1.3 [40] to calculate the variance components and significance levels of variation within a population and among populations.

#### 4.3.3. Isolation by Distance (IBD)

The correlation between genetic distance and geographic distance was assessed using IBDWS version 3.21 [41]. The significance of the analysis was examined with Mantel tests using the IBDWS software [42].

#### 4.3.4. Network Analysis

The evolutionary relationships among the haplotypes produced by the mitochondrial control region and *proPOx* intron were examined using TCS version 1.21 [43], and a statistical parsimony haplotype network was generated at the 95% connection limit.

#### 4.3.5. Scenario Testing

Scenario testing was applied separately to three different clusters. First, the populations La (from Louisiana, USA), Sa (from Saitama, Japan) and NJ (three populations from Nanjing, China combined) were set as cluster 1, and six scenarios were considered. An unsampled population was included in scenarios 3, 4, 5 and 6 to test for an unknown source of the second gene pool. For cluster 2, we classified the samples collected in China (excluding more admixture populations) into five populations/regions (R1: XYw, XYc, XG, BGt, XBv, DY, MAS, HF and CH; R2: JX, ZX and CQ; R3: YNL, CHL, QJ, NHL, LZL, XT, HHL, DTLs, DTL, YJ and NX; R4: WJ, CJr, SLt, NBp, PYL and NCyL; R5: WX, WXb, NT, JX, SH and NB). Generally, approximately 120 scenarios could be tested with five populations, but this number can be decreased using the hypotheses emerging from previous genetic studies and the known historical dates of the first record of invasion in a country [8]. Ultimately, five different scenarios were considered for cluster 2. Cluster 3 included the admixture populations (POP1, Region I; POP2, QJ; POP3, WJ, NBp and SLt combined; and POP4, WX and SH combined), and 19 scenarios were considered. The populations of Region I (POP1) were considered to be the origin of the other POPs (POP1, POP2 and POP3), and different introduction scenarios among the POPs were then compared.

The scenarios were tested using ABC analyses of demography in DIYABC software v1.0.4.46 beta [11]. Prior distributions were defined for three sets of analyses as follows: 1 < *t1* < *t2* < *t3* < *t4* < 100; 5 < *db* < 25; 1000 < *N* < 1,000,000; 1 < *Nf* < 10,000. The values of the demographic parameters were *t*, *db*, *N* and *Nf*. The values of the sample sizes that were measured in number of individuals were *N* and *Nf*. The values of times that were measured in number of generations were *db* and *t*. The default values of prior distributions of the different parameters for the mutation model of the X-linked and mitochondrial DNA sequences were used. Kimura’s two-parameter model of mutation was used [44] with 10% of invariant sites. The “one sample summary statistics” used were the number of haplotypes, the number of segregating sites and the mean of pairwise differences. The “two sample summary statistics” used were the means of pairwise differences (W) and (B). For the simulated data sets for each scenario, the default value (1,000,000) was created. Prior-scenario combinations were evaluated by performing a principal component analysis (PCA) using DIYABC. Because it can be useful to skip the logistic regression approach to obtain a rough estimate of the relative support of the scenarios through a direct estimate only [11], posterior probabilities (PP) of the most likely scenarios were identified using the direct estimate approach. The performances of parameter estimations were assessed by computing the relative bias and the relative root mean square error using all of the default values, including the number (500) of test data sets. Confidence in a scenario choice was evaluated by an option offered in DIYABC. The probability of a Type I error was estimated by calculating the proportion of times for which a given scenario did not have the highest PP among competing scenarios when it was actually the true scenario, and the probability of a type II error was estimated by determining the proportion of times for which a given scenario had the greatest PP when it was not actually the correct scenario.

## 5. Conclusions

The red swamp crayfish was most likely introduced into China from Japan from an unsampled source and subsequently mainly expanded and transferred to the middle and lower reaches and eventually the upper reaches of the Changjiang River. However, the results of this study also revealed that the dispersal pathways for *P. clarkii* might be much more complex than previously assumed. Human-mediated jump dispersal was an important dispersal pathway for *P. clarkii* in China. More sampling data and further analyses are needed to elucidate actual sources and further details of invasion routes.

## Supplementary Materials

Supplementary materials can be found at http://www.mdpi.com/1422-0067/16/07/14623/s1.
