# Supplementary material for: Inferring Invasion History of Red Swamp Crayfish (Procambarus clarkii) in China from Mitochondrial Control Region and Nuclear Intron Sequences
Source: Int J Mol Sci. 2015 Jun 29;16(7):14623–39. doi: 10.3390/ijms160714623 (PMC4519862; doi:10.3390/ijms160714623)
Supplement: Supplementary file 1 [file ijms-16-14623-s001.pdf]

Supplementary Information

haplotypes	SH	NB	JX	XYc	XYw	WXb	NT	XG	XBv	BGt	WX	WJ	MAS	CJr	CH	HF	DY	SLt	NBp	PYL	NCyl	NHL	YNL	XT	QJ	LZL	HHL	CHL	YJ	NX	DTL	DTLs	CQs	ZX	JY	Sa	Lo
Hap_d1	5		1			3	1					7	1		5		3	2															7			7	
Hap_d2	2	5	2	4	4	3	6	8	7	3	4		1	1	2		1	4	8	7	8	5	8	7	7		7	6	4	5	5	5		2	4	10	
Hap_d3		2	4		1	2				3				5		1	2	1				2		1		6		1	1	3		2			1		
Hap_d4		1																																			
Hap_d5				1																																	
Hap_d6				1																																	
Hap_d7				1																																	
Hap_d8					1																																
Hap_d9					1																																
Hap_d10						1																															
Hap_d11										1																											
Hap_d12											1																										
Hap_d13													3																								
Hap_d14													1																								
Hap_d15													1																								
Hap_d16													1																								
Hap_d17														1																							
Hap_d18																7																					
Hap_d19																		1																			
Hap_d20																											1										
Hap_d21																													2								
Hap_d22																															1						
Hap_d23																																1					
Hap_d24																																		4			
Hap_d25																																			1		
Hap_d26																																				1	
Hap_d27																																					1
Hap_d28																																					1
Hap_d29																																					1
Hap_d30																																					1
Hap_d31																																					1
Hap_d32																																					1
Hap_d33																																					1
Hap_d34																																					1
Hap_d35																																					1
Hap_d36																																					1
Hap_d37																																					1
Hap_d38																																					1
Hap_d39																																					1
Hap_d40																																					1
Hap_d41																																					1
Hap_d42																																					1
Hap_d43																																					1
Hap_d44																																					1
Hap_d45																																					1
Hap_d46																																					1

(a)

Figure S1. *Cont.*

haplotypes	SH	NB	JX	XYc	XYw	WXb	NT	XG	XBv	BGt	WX	WJ	MAS	CJr	CH	HF	DY	SLt	NBp	PYL	NCyl	NHL	YNL	XT	QJ	LZL	HHL	CHL	YJ	NX	DTL	DTLs	CQs	ZX	JY	Sa	Lo
Hap_i1	1	4	2		1	3	2	5	1	5	3	2	1	3	5	1			5	1			3	3		1	1	3	1	2	1	3	6	4	4	2	2
Hap_i2	1			3	3	2		2	5	2	1	5		2	2	5	3	5	1	1	3	4	3	3		1		2	1	1			2	2	2	1	2
Hap_i3	1			3		1	4				3		5												3		1	1	3	1	1	1				1	1
Hap_i4												1																								1	
Hap_i5																			1																	1	
Hap_i6																									1											1	
Hap_i7																																		1		1	
Hap_i8																																				1	
Hap_i9																																					4

(**b**)

Figure S1. Distribution of all haplotypes obtained. (**a**) Distribution of haplotypes in mitochondrial control region sequences of *P. clarkii*; (**b**) Distribution of haplotypes in *proPOx* intron sequences of *P. clarkii*. The sampling location codes see Table S1 in supplementary material.

Polymorphic sites (sites 8–692)

haplotype	8	35	36	37	55	62	84	93	94	139	151	166	272	284	306	319	342	385	451	556	620	621	630	644	648	649	655	656	667	683	684	686	692
hap_d1	A	G	G	A	C	A	C	A	A	C	A	C	A	T	T	C	A	G	A	A	C	A	T	A	A	C	A	T	A	A	T	A	T
hap_d2	.	.	A		T	.	.	.	.	.	G	.	.	.	.	T	G	.	.	.	T	G	.	.	.	T	.	C	.	.	.	.	.
hap_d3	.	.	A		T	.	.	.	.	.	G	.	.	.	.	T	G	.	.	.	T	G	.	.	.	T	.	C	.	.	.	.	.
hap_d4	.	.	A		T	.	.	.	.	.	G	.	.	.	.	T	G	.	.	.	T	G	.	.	.	T	.	C	.	.	.	.	.
hap_d5	.	.	A		T	.	.	.	.	.	G	.	.	.	.	T	G	.	.	.	T	G	.	G	.	T	.	C	G	G	G	G	.
hap_d6	.	.	A		T	G	.	G	G	.	G	.	.	.	.	T	G	.	.	.	T	G	.	G	.	T	.	C	G	G	G	G	.
hap_d7	.	.	A		T	G	.	G	G	.	G	.	.	.	.	T	G	.	.	.	T	G	.	.	.	T	.	C	.	.	.	.	.
hap_d8	.	.	A		T	.	.	.	.	.	G	.	.	.	.	T	G	.	.	.	T	G	.	.	.	T	.	C	.	.	.	.	.
hap_d9	.	.	A		T	.	.	.	.	.	G	.	.	.	.	T	G	.	.	.	T	G	.	.	.	T	.	C	.	.	.	.	.
hap_d10	.	.	A		T	.	.	.	.	.	G	.	.	.	.	T	G	.	.	.	T	G	.	.	.	T	.	C	.	.	.	.	.
hap_d11	.	.	A		T	.	.	.	.	.	G	.	.	.	.	T	G	.	.	.	T	G	.	.	.	T	.	C	.	.	.	.	.
hap_d12	.	.	.	.	.	.	.	.	.	.	.	.	.	.	.	.	.	.	.	.	.	.	.	.	.	.	.	.	.	.	.	.	.
hap_d13	.	.	.	.	.	.	.	.	.	.	.	.	.	.	.	.	.	.	.	.	.	.	.	.	.	.	.	.	.	.	.	.	.
hap_d14	.	C	A	.	T	.	.	.	.	.	G	.	.	.	.	T	G	.	.	.	T	G	.	.	.	T	.	C	.	.	.	.	.
hap_d15	.	.	.	.	.	.	.	.	.	.	.	.	.	.	.	.	.	.	.	.	.	.	.	.	.	.	.	.	.	.	.	.	.
hap_d16	.	.	.	.	.	.	.	.	.	.	.	.	.	.	.	.	.	.	.	.	.	.	.	.	.	.	.	.	.	.	.	.	.
hap_d17	.	.	A	.	T	.	.	.	.	.	G	.	.	.	.	T	G	.	.	.	T	G	.	.	.	T	.	.	.	.	.	.	.
hap_d18	.	.	.	.	.	.	.	.	.	.	.	.	.	.	.	.	.	.	.	.	.	.	.	.	.	.	.	.	.	.	.	.	.
hap_d19	.	.	A	.	T	.	.	.	.	.	T	.	.	.	.	T	G	.	.	.	T	G	.	.	.	T		C	.	.	.	.	.
hap_d20	.	.	A	.	T	.	.	.	.	.	G	.	.	.	.	T	G	.	.	.	T	G	.	.	.	T		C	.	.	.	.	.
hap_d21	.	.	A	.	T	.	.	.	.	.	G	.	.	.	.	T	G	.	.	.	T	G	.	.	.	T		C	.	.	.	.	.
hap_d22	.	.	A	.	T	.	.	.	.	.	G	.	.	.	.	T	G	.	.	.	T	G	.	.	.	T		C	.	.	.	.	.
hap_d23	.	.	A	.	T	.	.	.	.	.	G	T	.	.	.	T	G	.	.	.	T	G	.	.	.	.	.	.	.	.	.	.	.
hap_d24	.	.	.	.	.	.	.	.	.	.	.	.	.	.	.	.	.	.	.	.	.	.	.	.	.	.	.	.	.	.	.	.	.
hap_d25	.	.	.	G	.	.	.	.	.	.	.	.	.	.	.	.	.	.	.	.	.	.	.	.	.	.	.	.	.	.	.	.	.
hap_d26	.	.	.	.	.	.	.	.	.	.	.	.	.	.	.	.	.	.	.	.	.	.	.	.	.	.	.	.	.	.	.	.	.
hap_d27	.	.	.	.	.	.	.	.	.	.	G	.	.	.	.	.	G	A	.	.	.	.	.	.	G	.	.	.	.	.	.	.	.
hap_d28	.	.	.	.	.	.	T	.	.	.	G	.	.	.	.	.	G	A	.	.	.	.	.	.	G	.	.	.	.	.	.	.	.
hap_d29	.	.	.	.	.	.	.	.	.	.	G	.	.	.	.	.	.	A	.	.	.	.	.	.	G	.	.	.	.	.	.	.	.
hap_d30	.	.	.	.	.	.	.	.	.	.	G	.	.	.	.	.	G	A	.	.	.	.	.	.	G	.	.	.	.	.	.	.	.
hap_d31	.	.	.	.	.	.	.	.	.	.	G	.	G	.	.	.	G	A	.	.	.	.	.	.	G	.	G	.	.	.	.	.	.
hap_d32	.	.	.	.	.	.	.	.	.	A	G	.	.	.	.	.	G	A	.	.	.	.	.	.	G	.	.	.	.	.	.	.	.
hap_d33	.	.	.	.	.	.	.	.	.	.	.	.	.	.	C	.	.	.	.	.	.	.	.	.	.	.	.	C	.	.	.	.	.
hap_d34	G	.	.	.	.	.	.	.	.	.	G	.	.	.	.	.	G	A	.	.	.	.	.	.	G	.	.	.	.	.	.	.	.
hap_d35	.	.	.	.	.	.	T	.	.	.	G	.	.	.	.	.	G	A	.	.	.	.	.	.	G	.	.	.	.	.	.	.	.
hap_d36	.	.	.	.	.	.	.	.	.	.	G	.	.	.	.	.	G	A	.	.	.	.	C	.	G	.	G	.	.	.	.	.	.
hap_d37	.	.	.	.	.	.	.	.	.	.	.	.	.	.	C	.	.	.	.	.	.	.	.	.	.	.	.	C	.	.	.	.	C
hap_d38	.	.	.	.	.	.	.	.	.	.	G	.	.	.	.	.	G	.	G	.	.	.	.	.	.	.	.	.	.	.	.	.	.
hap_d39	.	.	.	.	.	.	.	.	.	.	G	.	.	.	.	.	G	A	.	.	.	.	.	.	G	.	.	.	.	.	.	.	.
hap_d40	.	.	.	.	.	.	.	.	.	.	G	.	.	.	.	.	G	.	G	T	.	.	.	.	.	.	.	.	.	.	.	.	.
hap_d41	.	.	.	.	.	.	.	.	.	.	G	.	.	.	.	.	.	A	.	.	.	.	.	.	G	.	.	.	.	.	.	.	.
hap_d42	.	.	.	.	.	.	.	.	.	.	G	.	.	A	.	.	G	.	G	.	.	.	.	.	.	.	G	.	.	.	.	.	.
hap_d43	.	.	.	.	.	.	.	.	.	.	G	.	.	.	.	.	G	.	.	.	.	.	.	.	G	.	.	.	.	.	.	.	.
hap_d44	.	.	.	.	.	.	.	.	.	.	G	.	.	.	.	.	G	A	.	.	.	.	.	.	G	.	.	.	.	.	.	.	.
hap_d45	.	.	.	.	.	.	.	.	.	.	G	.	.	.	.	.	G	A	.	.	.	.	.	.	G	.	.	.	.	.	.	.	.
hap_d46	.	.	.	.	.	.	.	.	.	.	G	.	.	.	.	.	G	.	G	.	.	.	.	.	G	.	.	.	.	.	.	.	.

Polymorphic sites (sites 701–964)

haplotype	701	704	710	732	757	761	789	802	803	829	846	847	850	852	854	856	862	863	868	872	876	882	883	888	889	894	899	903	904	906	943	964
hap_d1	T	G	T	T	A	T	T	T	A	G	G	T	C	T	C	A	T	A	A	G	A	G	T	A	T	T	T	T	A	A	A	T
hap_d2	.	A	.	.	.	.	.	.	G	A	.	.	T	.	G	G	.	.	.	A	.	A	.	T	A	C	C	A	T	T	.	.
hap_d3	.	A	.	.	.	.	.	.	G	A	.	.	T	.	G	G	.	.	.	A	.	A	.	T	A	C	C	A	T	T	.	.
hap_d4	.	A	.	.	.	.	.	.	G	A	.	.	T	.	G	G	.	.	.	A	.	A	.	T	A	C	C	A	T	T	.	.
hap_d5	.	A	.	.	G		G	G	G	A	.	.	T	.	G	G	.	.	.	A	.	A	.	T	A	C	C	A	T	T	.	.
hap_d6	.	A	.	.	G		G	G	G	A	.	.	T	.	G	G	.	.	.	A	.	A	.	T	A	C	C	A	T	T	.	.
hap_d7	.	A	.	.	.	.	.	.	G	A	.	.	T	.	G	G	.	.	.	A	.	A	.	T	A	C	C	A	T	T	.	.
hap_d8	.	A	.	.	.	.	.	.	G	A	.	.	T	.	G	G	.	.	.	A	.	A	.	T	A	C	C	A	T	T	.	.
hap_d9	.	A	.	.	.	.	.	.	G		.	.	T	.	G	G	.	.	.	A	.	A	.	T	A	C	C	A	T	T	.	.
hap_d10	.	A	.	.	.	.	.	.	G	A	.	.	T	.	G	G	.	.	.	A	.	A	.	T	A	C	C	A	T	T	.	.
hap_d11	.	A	.	.	.	.	.	.	G	A	.	.	T	.	G	G	.	.	.	A	.	A	.	T	A	C		A	T	T	.	.
hap_d12	.	.	.	.	.	.	.	.	.	.	.	.	.	.	.	.	.	.	.	.	.	.	.	.	.	.	.	.	.	.	.	.
hap_d13	.	.	.	.	.	.	.	.	.	.	.	.	.	.	.	.	.	.	.	.	.	.	.	.	.	.	.	.	.	.	.	.
hap_d14	.	A	.	.	.	.	.	.	G	A	.	.	T	.	G	G	.	.	.	A	.	A	.	T	A	C	C	A	T	T	.	.
hap_d15	.	.	.	.	.	.	.	.	.	.	.	.	.	.	.	.	.	.	C	.	.	.	.	.	.	.	.	.	.	.	.	.
hap_d16	.	.	.	.	.	.	.	.	.	.	.	.	.	.	.	.	.	.	.	.	.	.	.	.	.	.	.	.	.	.	.	.
hap_d17	.	A	.	.	.	.	.	.	G	A	.	.	T	.	G	G	.	.	.	A	.	A	.	T	A	C	C	A	T	T	.	.
hap_d18	.	.	.	.	.	.	.	.	.	.	.	.	.	.	.	.	.	.	.	.	.	.	.	.	.	.	.	.	.	.	.	.
hap_d19	.	A	.	.	.	.	.	.	G	A	.	.	T	.	G	G	.	.	.	A	.	A	.	T	A	C	C	A	T	T	.	.
hap_d20	.	A	.	.	.	.	.	.	G	A	.	G	T	.	G	G	.	.	.	A	.	A	.	T	A	C	C	A	T	T	.	.
hap_d21	.	A	.	.	.	.	.	.	G	A	.	.	T	.	G	G	.	.	.	A	.	A	.	T	A	C	C	A	T	T	.	.
hap_d22	.	A	.	.	.	.	.	.	G	A	.	.	T	.	G	G	.	.	.	A	.	A	.	T	A	C	C	A	T	T	.	.
hap_d23	.	A	.	.	.	.	.	.	G	A	.	.	T	.	G	G	.	.	.	A	.	A	.	T	A	C	C	A	T	T	.	.
hap_d24	.	.	.	.	.	.	.	.	.	.	.	.	.	.	.	.	.	.	.	.	.	.	.	.	.	.	.	.	.	.	.	G
hap_d25	.	.	.	.	.	.	.	.	.	.	.	.	.	.	.	.	.	.	.	.	.	.	.	.	.	.	.	.	.	.	.	.
hap_d26	.	.	.	.	.	.	.	.	.	.	.	.	.	.	.	.	.	.	.	.	.	.	.	.	.	C	C	A	T	T	.	.
hap_d27	C	A	.	C	.	.	.	.	.	A	.	.	T	.	G	G	.	G	.	A	G	A	C	T	A	.	C	A	T	T	.	.
hap_d28	C	A	.	C	.	.	.	.	.	A	.	.	T	.	G	G	.	G	.	A	.	A	C	T	A	C	C	A	T	T	.	.
hap_d29	C	A	.	C	.	.	.	.	.	A	.	.	T	.	G	G	.	G	.	A	.	A	C	T	A	C	C	A	T	T	.	.
hap_d30	C	A	.	C	.	.	.	.	.	A	.	.	T	.	G	G	.	G	.	A	.	A	C	T	A	C	C	A	T	T	.	.
hap_d31	.	A	.	.	.	.	.	.	.	A	.	.	T	.	G	G	C	G	.	A	.	A	C	T	A	.	C	A	T	T	.	.
hap_d32	C	A	C	C	.	.	.	.	.	A	.	.	T	.	G	G	.	G	.	A	.	A	C	T	A	.	C	G	T	T	.	.
hap_d33	C	A	.	C	.	.	.	.	.	A	.	.	T	.	G	G	.	G	.	A	.	A	C	T	A	C	C	A	T	T	.	.
hap_d34	C	A	.	C	.	.	.	.	.	A	.	.	T	.	G	G	.	G	.	A	.	A	C	T	A	C	C	A	T	T	.	.
hap_d35	C	A	.	C	.	.	.	.	.	A	.	.	T	.	G	G	.	G	.	A	.	A	C	T	A	C	C	A	T	T	.	.
hap_d36	.	A	.	.	.	.	.	.	.	A	.	.	T	.	G	G	.	G	.	A	G	A	C	T	A	.	C	A	T	T	.	.
hap_d37	.	.	.	.	.	.	.	.	.	.	.	.	.	.	.	.	.	.	.	.	.	.	.	T	A	C	C	A	T	T	.	.
hap_d38	.	.	.	.	.	.	.	.	.	A	A	.	T	C	G	G	.	G	.	A	.	A	C	T	A	.	C	A	T	T	G	.
hap_d39	C	A	.	C	.	.	.	.	.	A	.	.	T	.	G	G	.	G	.	A	.	A	C	T	A	C	C	A	T	T	.	.
hap_d40	.	A	.	.	.	A	.	.	.	A	A	.	T	C	G	G	.	G	.	A	.	A	C	T	A	.	C	A	T	T	G	.
hap_d41	C	A	.	C	.	.	.	.	.	A	.	.	T	.	G	G	.	G	.	A	G	A	C	T	A	.	C	A	T	T	.	.
hap_d42	.	A	.	.	.	A	.	.	.	A	A	.	T	C	G	G	.	G	.	A	.	A	C	T	A	.	C	A	T	T	G	.
hap_d43	C	A	.	C	.	.	.	.	.	A	.	.	T	.	G	G	.	G	.	A	.	A	C	T	A	C	C	A	T	T	.	.
hap_d44	C	A	.	C	.	.	.	.	.	A	.	.	T	.	G	G	.	G	.	A	.	A	C	T	A	C	C	A	T	T	.	.
hap_d45	C	A	.	C	.	.	.	.	.	A	.	.	T	.	G	G	.	G	.	A	.	A	C	T	A	C	C	A	T	T	.	.
hap_d46	C	A	.	.	.	A	.	.	.	A	A	.	T	C	G	G	.	G	.	A	.	A	C	T	A	.	C	A	T	T	G	.

(**a**)

Polymorphic sites

haplotype	19	55	58	60	61	62	63	64	74	80	82	87	167	170	175	229
hap_i1	G	T	C	T	A	A	T	T	T	C	T	C	C	A	C	A
hap_i2	.	.	.	.	.		.	.	.	.	.	.	.	.	.	T
hap_i3	T	.	T	.	.	T	.	.	.	.	.	.	.	.	.	.
hap_i4	.	.	.	.	.	.	.	.	.	.	.	.	.	.	.	.
hap_i5	.	.	.	.	.	.	.	.	C	.	.	.	.	.	.	.
hap_i6	T	A	A	G	C	A	C	A	.	G	A	.	.	.	.	.
hap_i7	.	.	.	A	.	.	.	.	A	.	.	.	.	.	A	T
hap_i8	.	.	.	.	.	.	.	.	.	.	.	.	T	T	.	.
hap_i9	.	.	.	.	.	.	.	.	.	.	.	A	.	.	.	.

(**b**)

Figure S2. Summary of polymorphism in the mtDNA control region (**a**) and *proPO*x intron (**b**) in *P. clarkii*. The numbers of the polymorphic sites are indicated at the top.

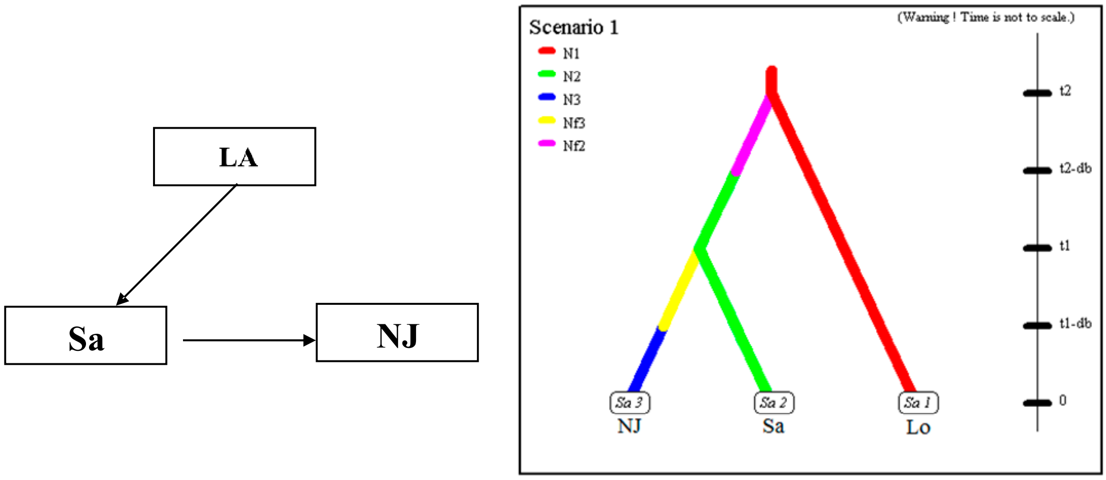

Scenario 1

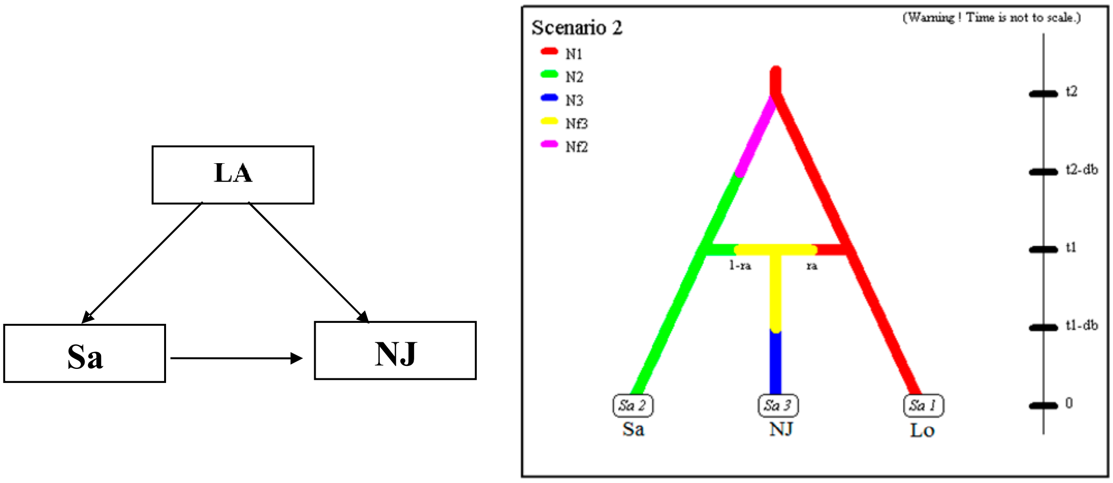

Scenario 2

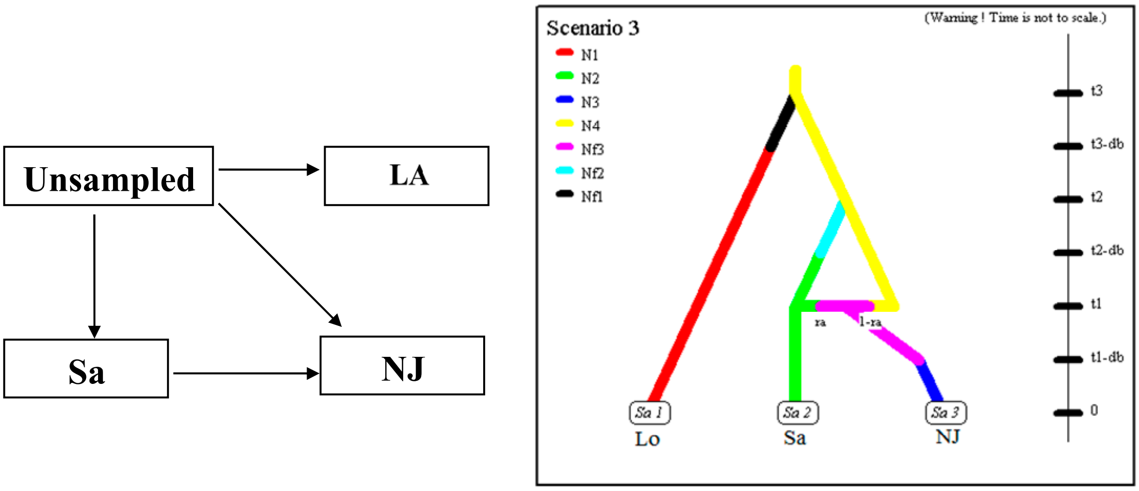

Scenario 3

Figure S3. *Cont.*

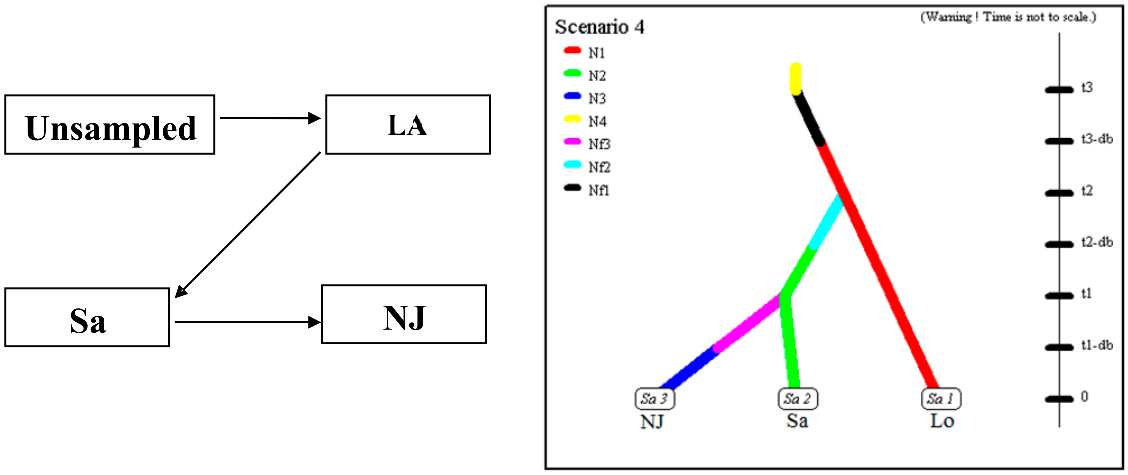

Scenario 4

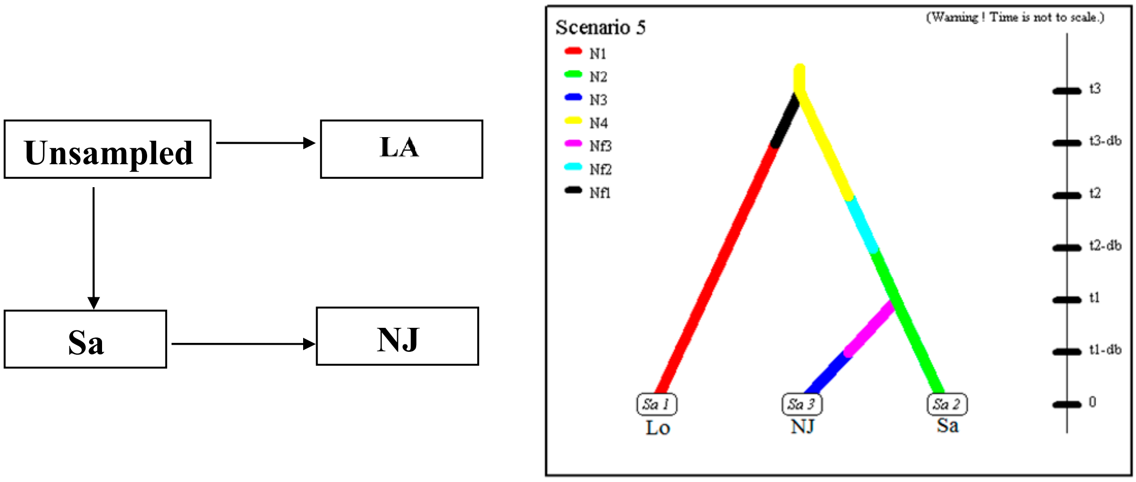

Scenario 5

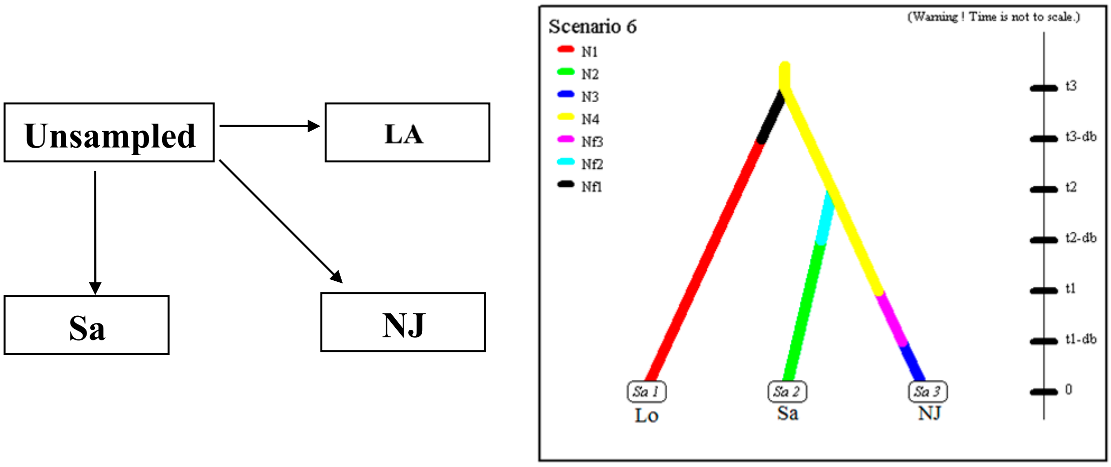

Scenario 6

Figure S3. *Cont.*

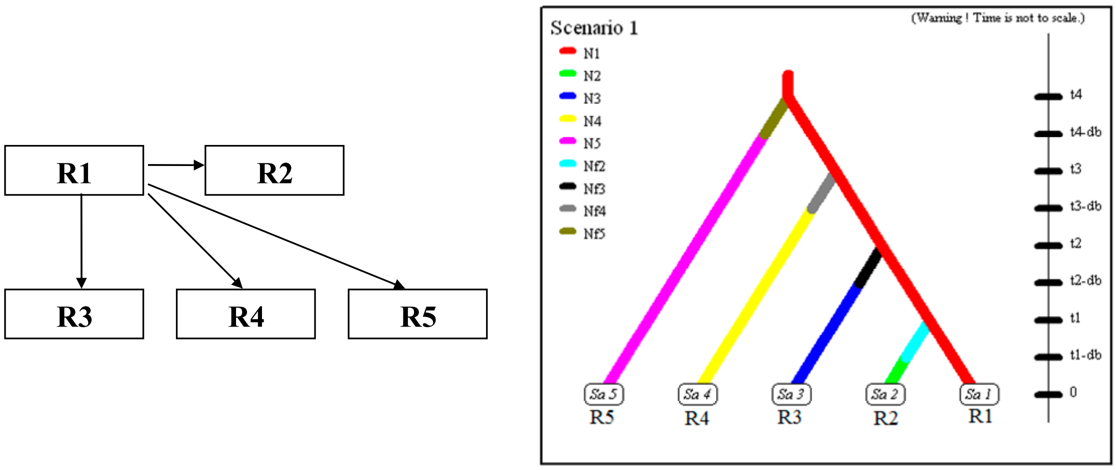

Scenario 7

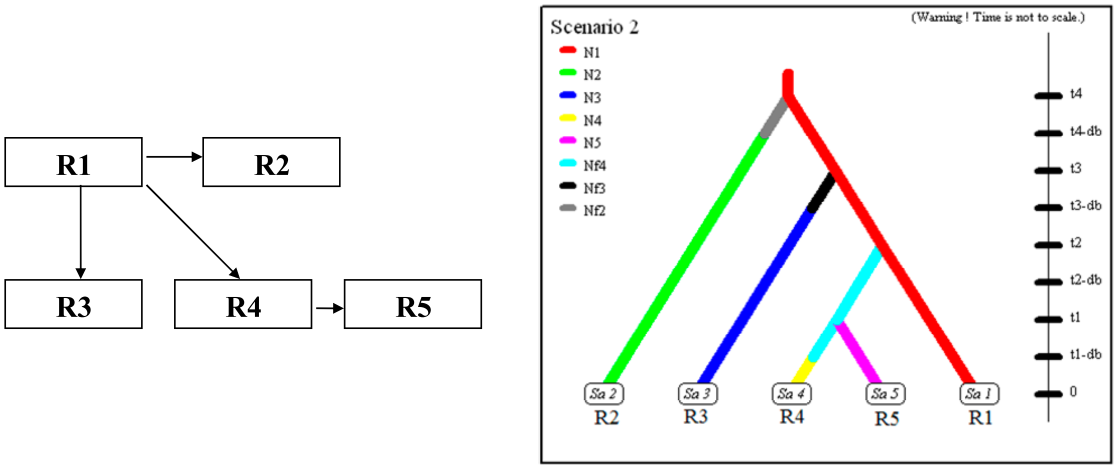

Scenario 8

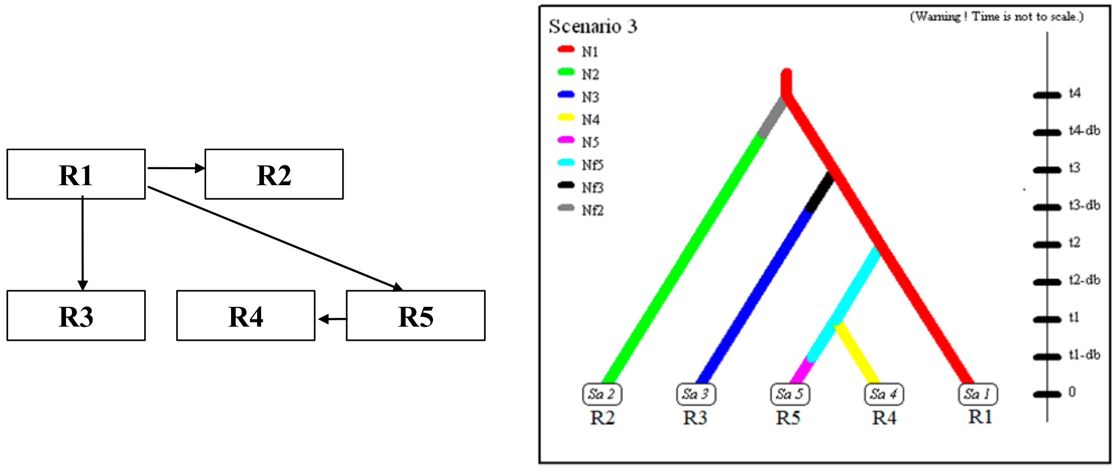

Scenario 9

Figure S3. *Cont.*

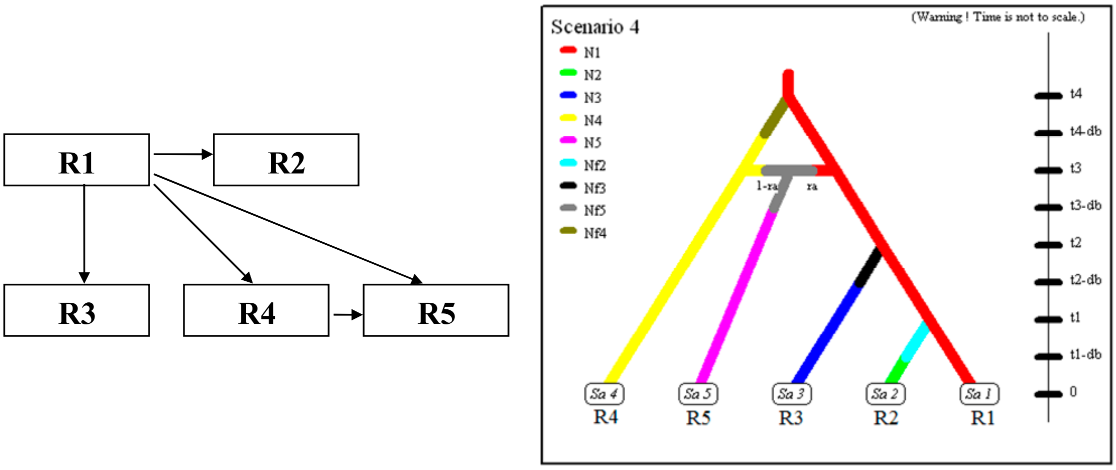

Scenario 10

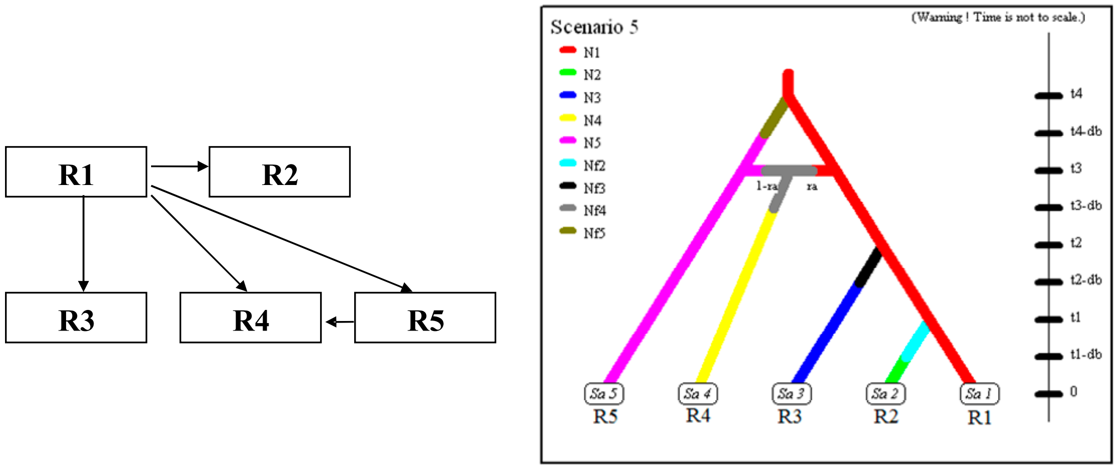

Scenario 11

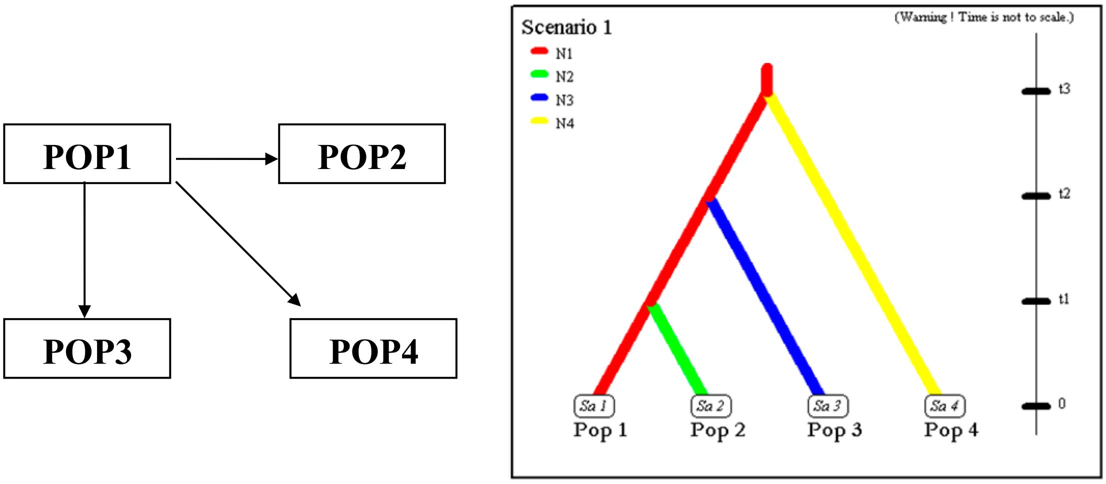

Scenario 12

Figure S3. *Cont.*

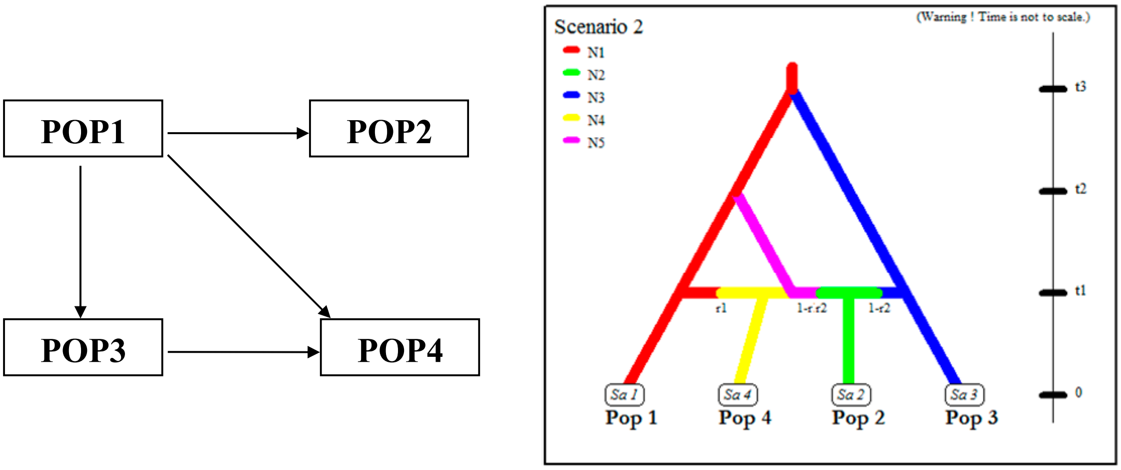

Scenario 13

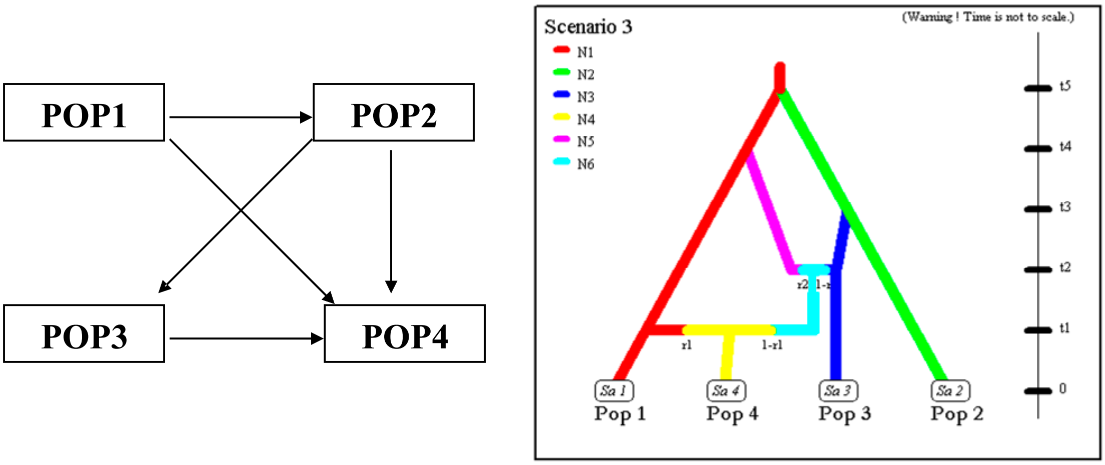

Scenario 14

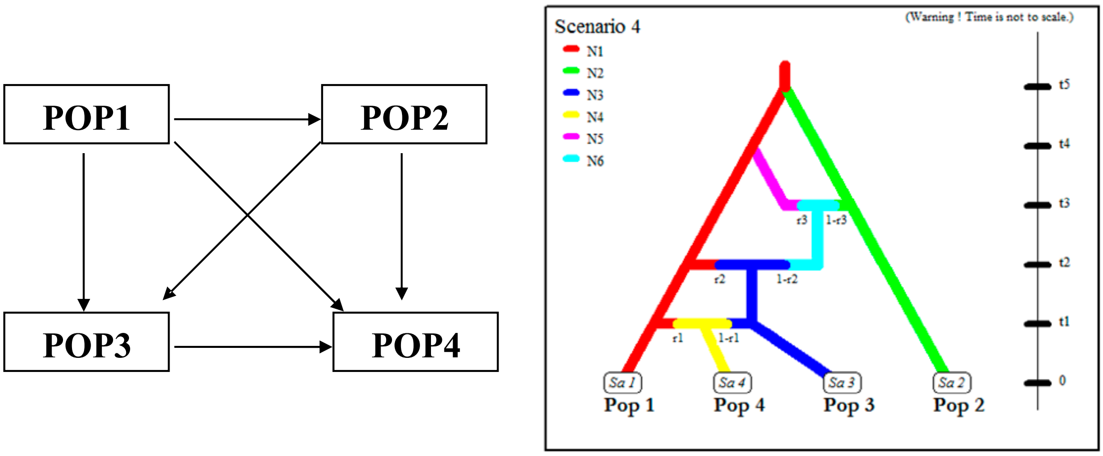

Scenario 15

Figure S3. *Cont.*

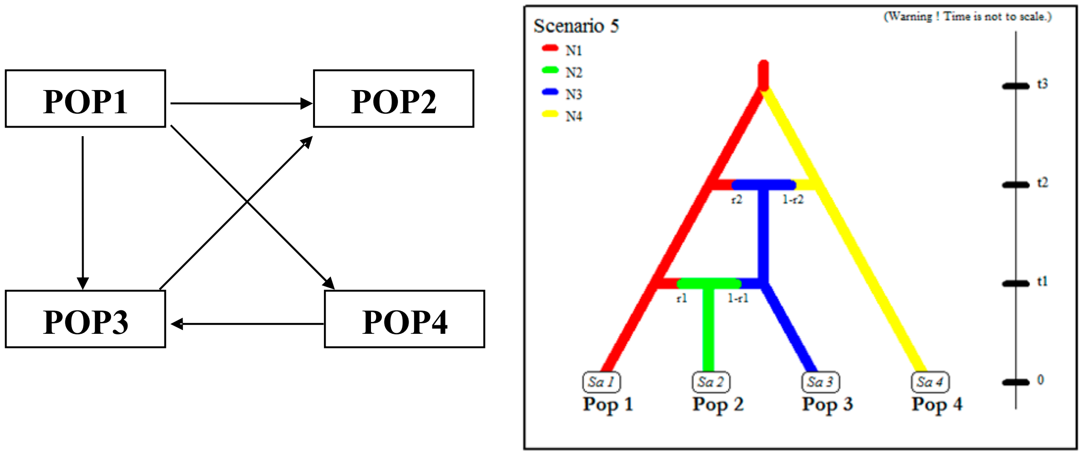

Scenario 16

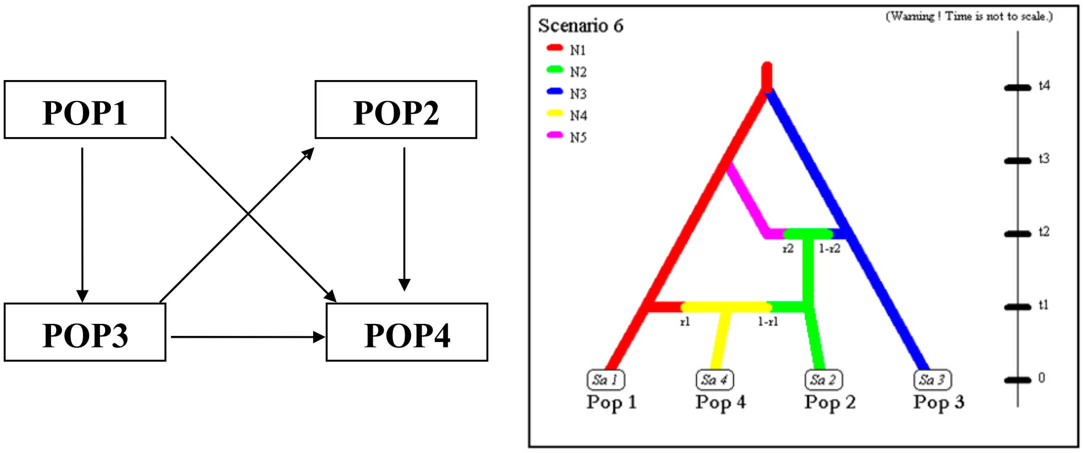

Scenario 17

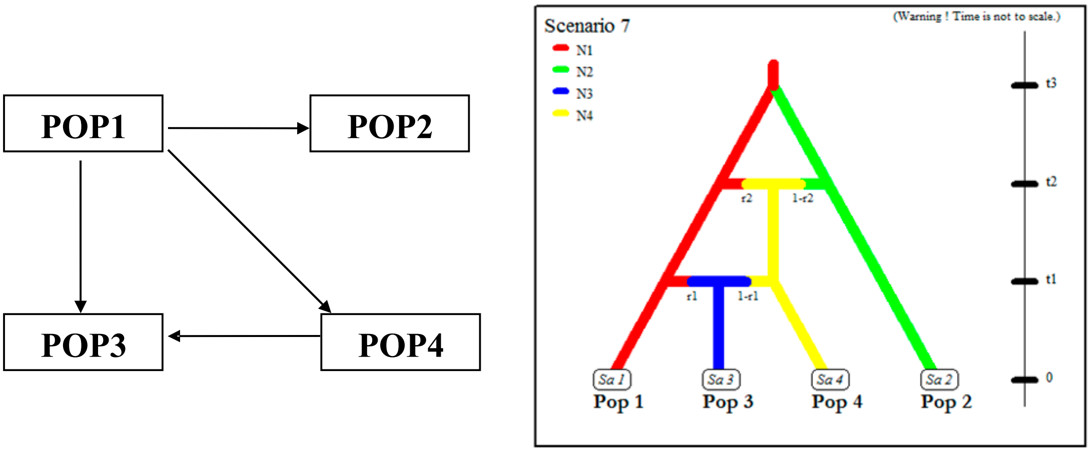

Scenario 18

Figure S3. *Cont.*

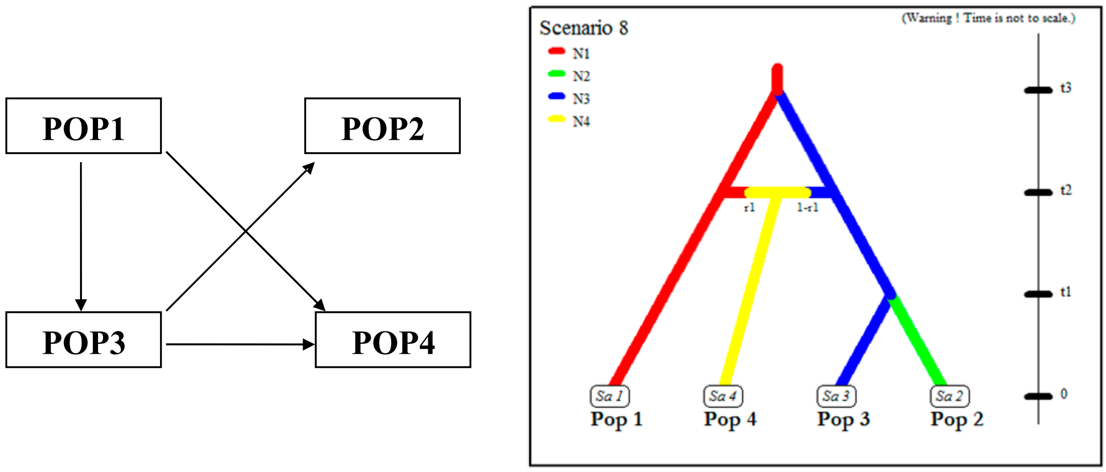

Scenario 19

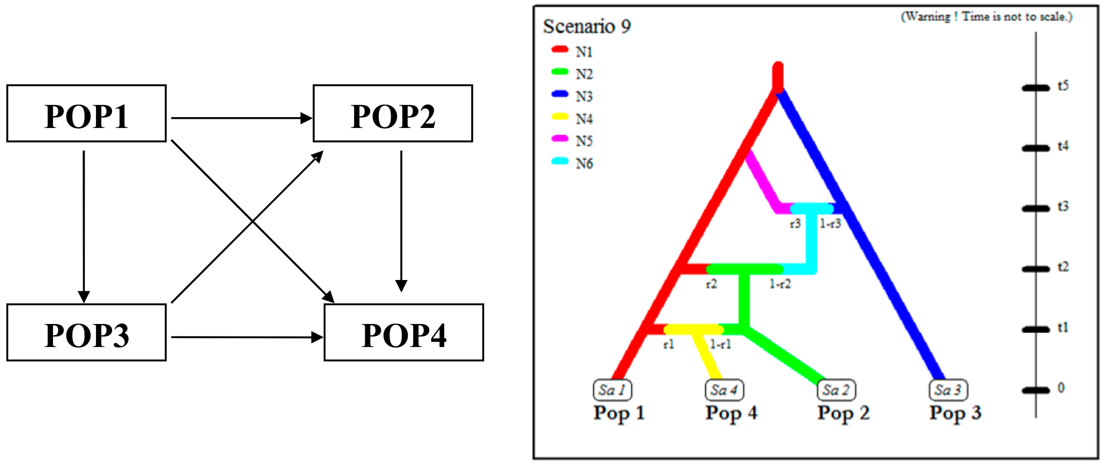

Scenario 20

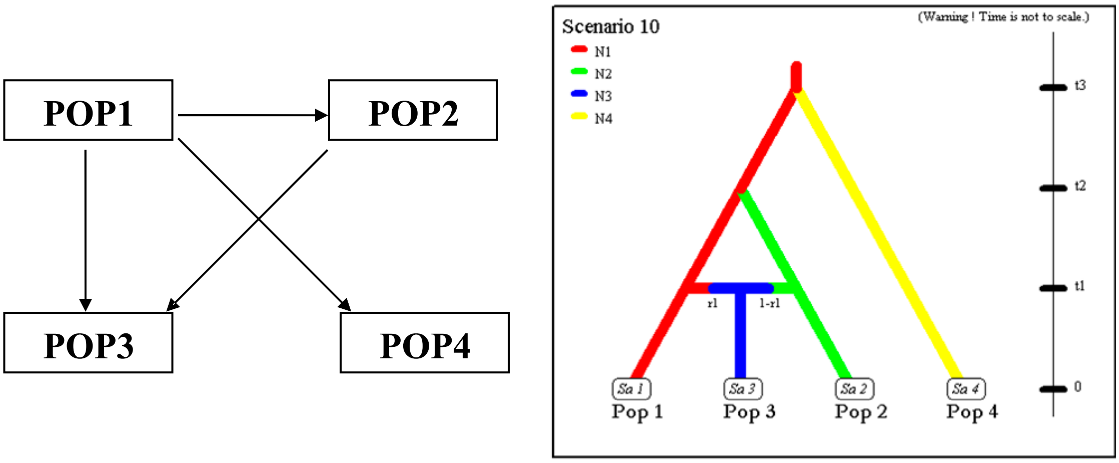

Scenario 21

Figure S3. *Cont.*

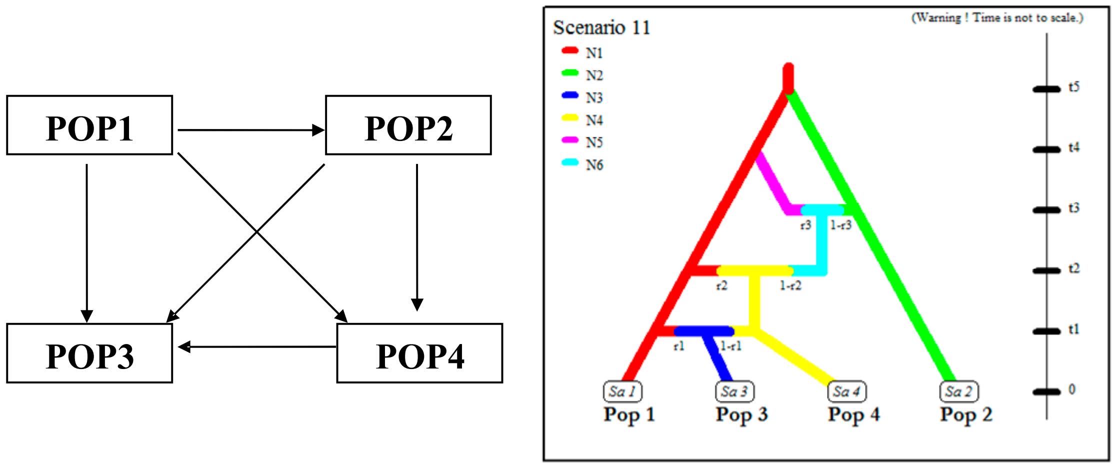

Scenario 22

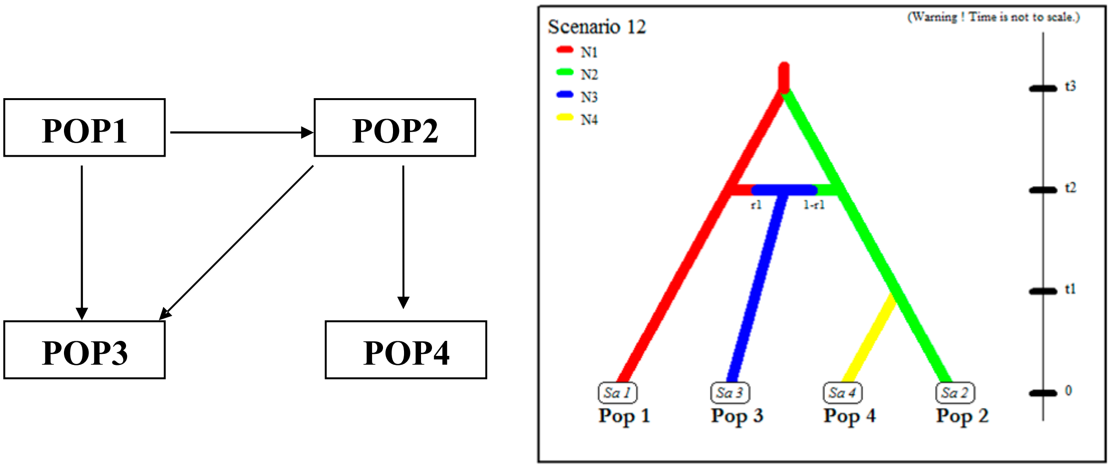

Scenario 23

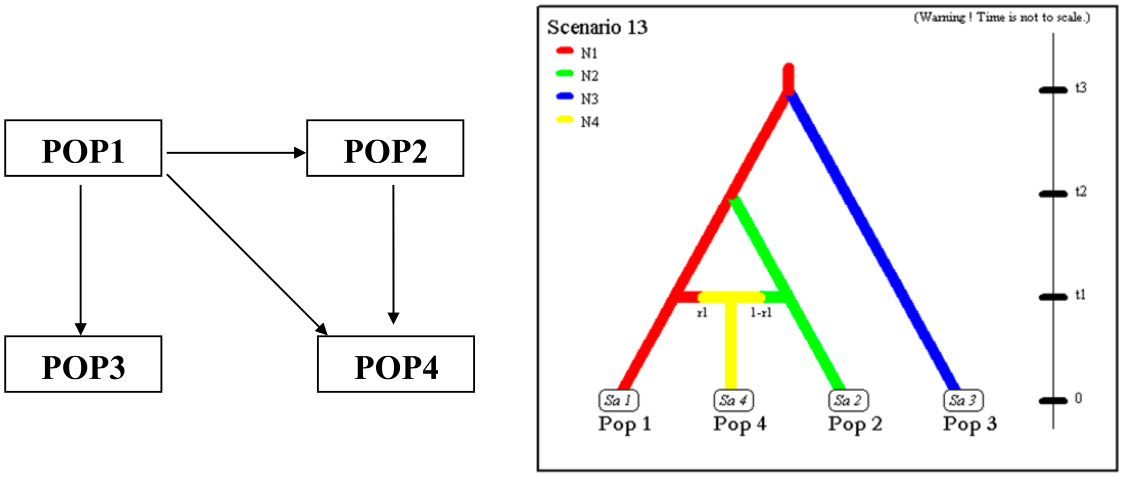

Scenario 24

Figure S3. *Cont.*

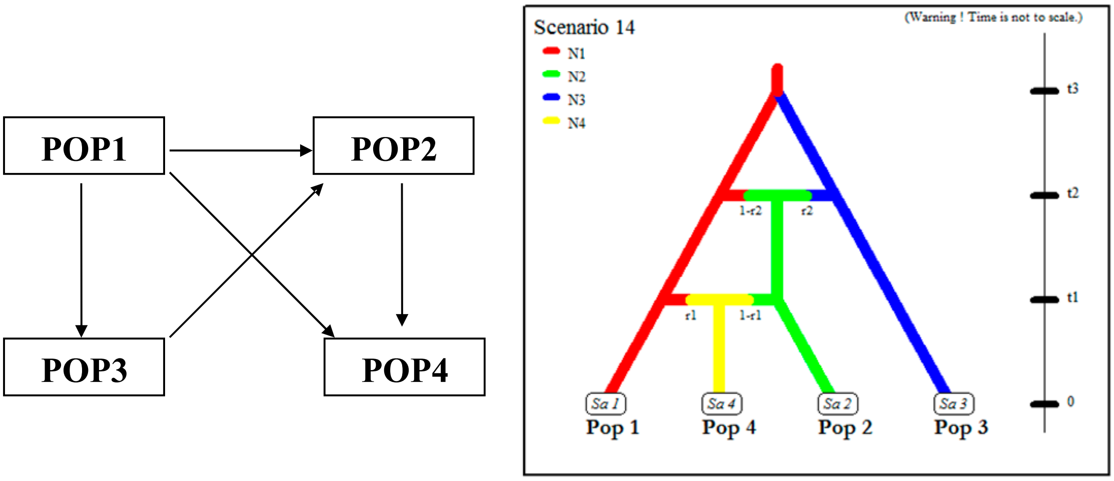

Scenario 25

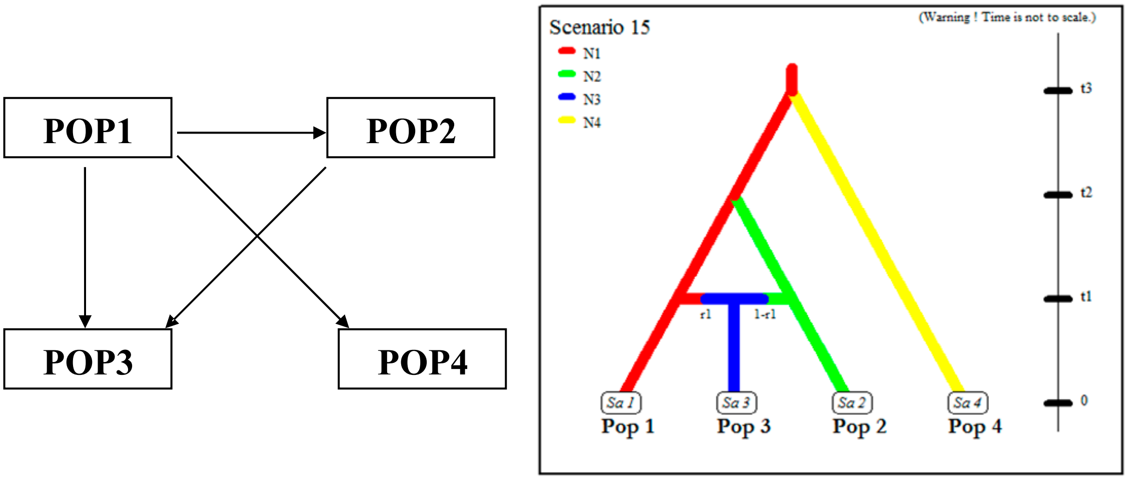

Scenario 26

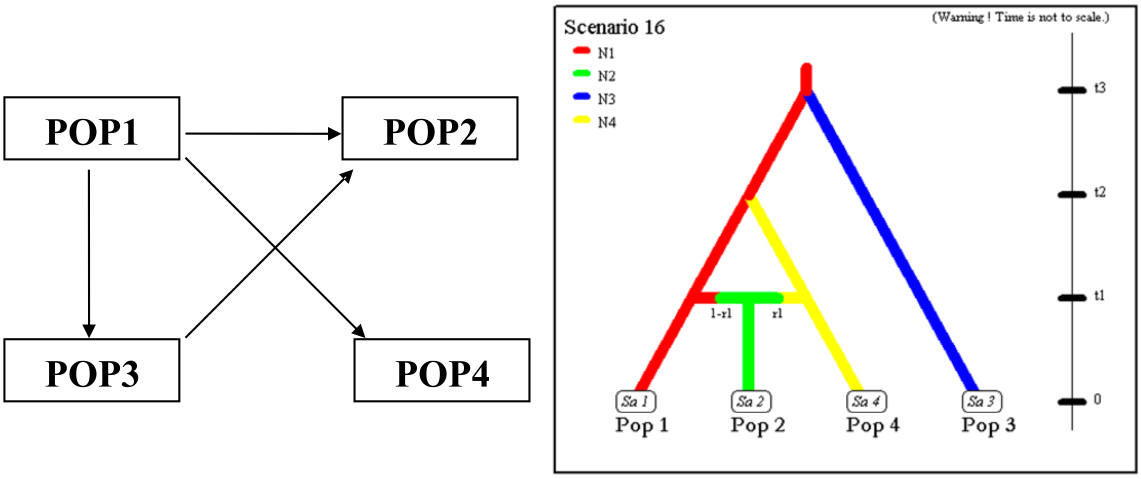

Scenario 27

Figure S3. *Cont.*

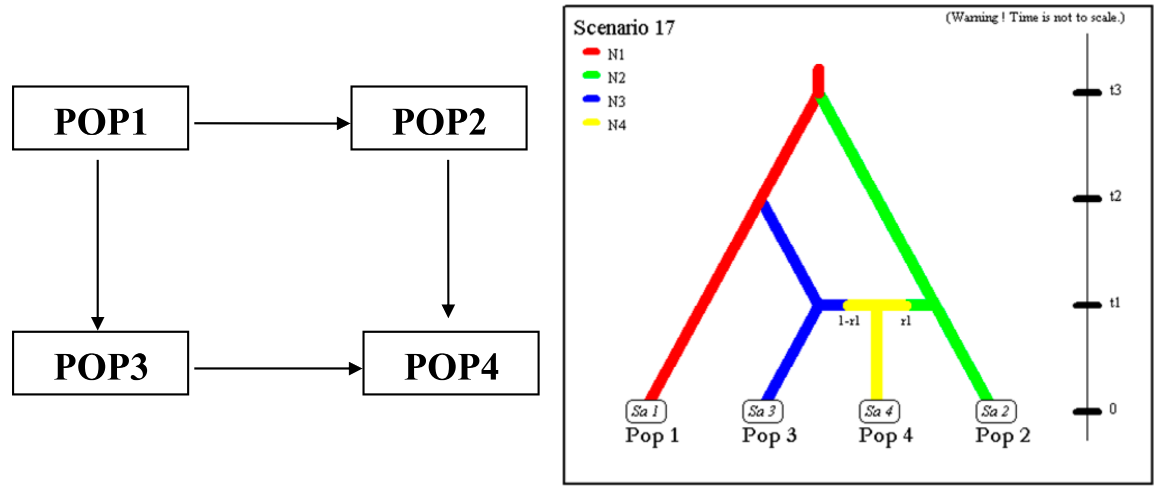

Scenario 28

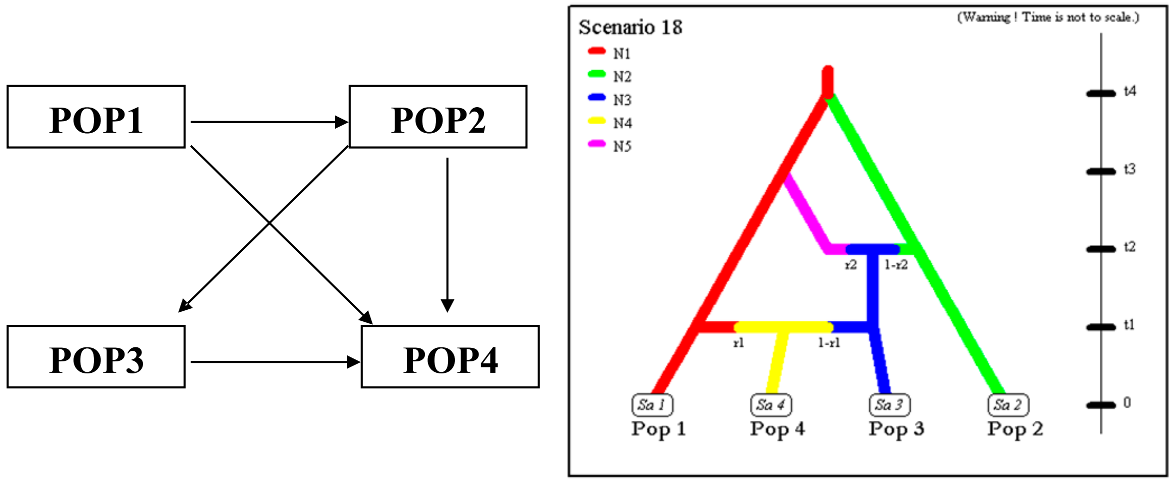

Scenario 29

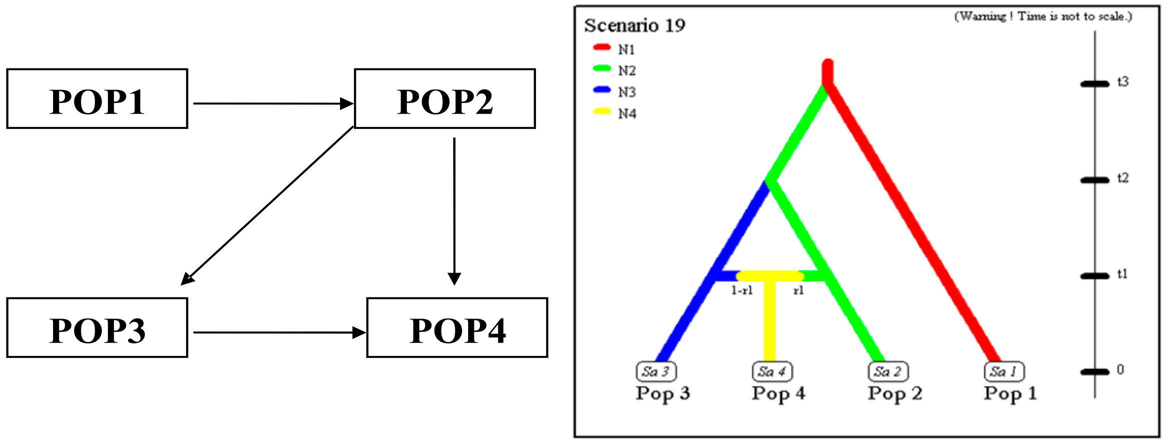

Scenario 30

Figure S3. Distinct scenarios tested using the software DIYABC and schematic representation of the competing scenarios considered for the inference of the invasion routes of *P. clarkii* in China. Scenario 1 to 6: hypothesized for Cluster 1; Scenario 6 to 11: hypothesized for Cluster 2; Scenario 12 to 30: hypothesized for Cluster 3.

Table S1. Analysis of molecular variance within and among *P. clarkii* populations by mitochondrial control region and *proPOx* intron analysis.

Source of Variance	*d.f.*	Sum of Squares	Variance Components	Percentage of Variation (%)
Control region analysis				
Among populations	36	1732.506	5.54030 Va	54.09
Within populations	254	1194.766	4.70181 Vb	45.91
Total	290	2926.766	10.24211	
ProPOx intron analysis				
Among populations	36	208.138	0.76471 Va	30.45
Within populations	159	277.729	1.74673 Vb	69.55
Total	195	485.867	2.51144	

Table S2. Relative posterior probabilities with 95% confidence intervals for all scenarios compared using Approximate Bayesian Computation, and results of type I and type II error rates. Power analyses were based on 100 datasets simulated under the different scenarios. Calculations were not performed for scenarios 12 to 30, as no scenario had higher posterior probability.

Scenario	Posterior Probability	Type I Error Rate	Type II Error Rate
1	0.0000 [0.0000–0.0000]		
2	0.0000 [0.0000–0.0000]		
3	0.1800 [0.0000–0.5168]		
4	0.1300 [0.0000–0.4248]		
5	0.6900 [0.2846–1.0000]	0.712	Mean: 0.172 (Min.: 0.000; Max.: 0.288)
6	0.0000 [0.0000–0.0000]		
7	0.0300 [0.0000–0.1795]		
8	0.4700 [0.0325–0.9075]	0.178	Mean: 0.158 (Min.:0.028; Max.: 0.202)
9	0.3600 [0.0000–0.7807]		
10	0.0800 [0.0000–0.3178]		
11	0.0600 [0.0000–0.2682]		
12	0.0400 [0.0000–0.2118]		
13	0.0700 [0.0000–0.2936]		
14	0.0500 [0.0000–0.2410]		
15	0.0500 [0.0000–0.2410]		
16	0.0500 [0.0000–0.2410]		
17	0.0400 [0.0000–0.2118]		
18	0.0200 [0.0000–0.1427]		
19	0.0400 [0.0000–0.2118]		
20	0.1200 [0.0000–0.4048]		
21	0.0400 [0.0000–0.2118]		
22	0.0400 [0.0000–0.2118]		
23	0.0600 [0.0000–0.2682]		
24	0.0600 [0.0000–0.2682]		
25	0.0600 [0.0000–0.2682]		
26	0.0500 [0.0000–0.2410]		

Table S2. *Cont.*

Scenario	Posterior Probability	Type I Error Rate	Type II Error Rate
27	0.0400 [0.0000–0.2118]		
28	0.0500 [0.0000–0.2410]		
29	0.0500 [0.0000–0.2410]		
30	0.0700 [0.0000–0.2936]		
